# Preventive CCL2/CCR2 Axis Blockade Suppresses Osteoclast Activity in a Mouse Model of Rheumatoid Arthritis by Reducing Homing of CCR2^hi^ Osteoclast Progenitors to the Affected Bone

**DOI:** 10.3389/fimmu.2021.767231

**Published:** 2021-12-03

**Authors:** Darja Flegar, Maša Filipović, Alan Šućur, Antonio Markotić, Nina Lukač, Dino Šisl, Marina Ikić Matijašević, Zrinka Jajić, Tomislav Kelava, Vedran Katavić, Nataša Kovačić, Danka Grčević

**Affiliations:** ^1^ Department of Physiology and Immunology, University of Zagreb School of Medicine, Zagreb, Croatia; ^2^ Laboratory for Molecular Immunology, Croatian Institute for Brain Research, University of Zagreb School of Medicine, Zagreb, Croatia; ^3^ Center for Clinical Pharmacology, University Clinical Hospital Mostar, Mostar, Bosnia and Herzegovina; ^4^ Department of Physiology, School of Medicine, University of Mostar, Mostar, Bosnia and Herzegovina; ^5^ Department of Anatomy, University of Zagreb School of Medicine, Zagreb, Croatia; ^6^ Department of Clinical Immunology, Rheumatology and Pulmology, Sveti Duh University Hospital, Zagreb, Croatia; ^7^ Department of Rheumatology, Physical Medicine and Rehabilitation, Clinical Hospital Center Sestre Milosrdnice, University of Zagreb School of Medicine, Zagreb, Croatia

**Keywords:** inflammation, myeloid cells, osteoclasts, chemokines, mouse arthritis, human arthritis

## Abstract

Detailed characterization of medullary and extramedullary reservoirs of osteoclast progenitors (OCPs) is required to understand the pathophysiology of increased periarticular and systemic bone resorption in arthritis. In this study, we focused on identifying the OCP population specifically induced by arthritis and the role of circulatory OCPs in inflammatory bone loss. In addition, we determined the relevant chemokine axis responsible for their migration, and targeted the attraction signal to reduce bone resorption in murine collagen-induced arthritis (CIA). OCPs were expanded in periarticular as well as circulatory compartment of arthritic mice, particularly the CCR2^hi^ subset. This subset demonstrated enhanced osteoclastogenic activity in arthritis, whereas its migratory potential was susceptible to CCR2 blockade *in vitro*. Intravascular compartment of the periarticular area contained increased frequency of OCPs with the ability to home to the arthritic bone, as demonstrated *in vivo* by intravascular staining and adoptive transfer of splenic LysMcre/Ai9 tdTomato-expressing cells. Simultaneously, CCL2 levels were increased locally and systemically in arthritic mice. Mouse cohorts were treated with the small-molecule inhibitor (SMI) of CCR2 alone or in combination with methotrexate (MTX). Preventive CCR2/CCL2 axis blockade *in vivo* reduced bone resorption and OCP frequency, whereas combining with MTX treatment also decreased disease clinical score, number of active osteoclasts, and OCP differentiation potential. In conclusion, our study characterized the functional properties of two distinct OCP subsets in CIA, based on their CCR2 expression levels, implying that the CCR2^hi^ circulatory-like subset is specifically induced by arthritis. Signaling through the CCL2/CCR2 axis contributes to OCP homing in the inflamed joints and to their increased osteoclastogenic potential. Therefore, addition of CCL2/CCR2 blockade early in the course of arthritis is a promising approach to reduce bone pathology.

## Introduction

Rheumatoid arthritis (RA) is a chronic, systemic, autoimmune inflammatory disease affecting 1% of the population worldwide (1). Inflammation-induced activation of osteoclasts, specialized bone resorbing cells, mediates bone loss in RA seen as marginal erosions followed by articular destruction, periarticular osteopenia, and generalized osteoporosis (2–4). Joint destruction eventually progresses, resulting in irreversible deformity, so prompt treatment is required at the disease onset to prevent disability. Currently available therapeutic strategies mostly address inflammatory/autoimmune component, whereas direct targeting of osteoclast differentiation and activity is noticeably disregarded.

Multinuclear bone resorbing osteoclasts develop from myeloid lineage progenitors in the bone marrow and among peripheral blood monocytes, which can generate macrophages and dendritic cells as well (5, 6). Recently, single-cell transcriptome technologies and lineage tracing studies have indicated additional levels of osteoclast progenitor (OCP) plasticity (7). The current concept suggests that osteoclasts arise from two sources of progenitors, the embryonic yolk sac-derived erythromyeloid extramedullar progenitors and hematopoietic stem cell-derived myeloid progenitors (8). Furthermore, the data from cell-fate analyses indicated the possibility of cell–cell fusion between these two lineages (9). During inflammation-induced bone resorption, there is an established osteoclast activity in places far from the hematopoietically active bone marrow. It is, therefore, clear that OCPs must migrate to bone surfaces either within a bone or *via* bloodstream, to engage in bone resorption (10, 11). However, it remains unclear whether osteoclasts derive predominantly from circulating monocytes or bone marrow-resident progenitors (12). A recent review by Yahara et al. suggests that additional studies are needed to reveal the mechanisms that orchestrate the mobilization of circulating OCPs from the extramedullary organs to bone in pathologic or homeostatic conditions (9).

OCP cells express multiple CC- and CXC-chemokine receptors, and are subject to chemokine signaling, as confirmed by research in mice and humans (6, 13–18). Despite numerous studies that have established the phenotypic markers of circulatory and bone marrow OCP subpopulations, their migration patterns and functional contribution to inflammatory diseases are not clearly defined (19). Chemokine signaling inhibition in mouse models of inflammatory diseases gave promising results (20–22), but so far there is limited or no success in clinical trials (23–27). Possible explanations lie not only in the robustness of the chemokine network, but also in the disease stage at treatment administration. It is reasonable to suspect that only early stage can respond to chemokine treatment, since in fully developed disease, the inflammatory infiltrate is already formed from migrated cells. In addition, clinical research asserts the importance of early diagnosis and intervention, suggesting that there might be a short period immediately upon the onset of RA when it is most susceptible to treatment (28, 29).

In this study, we focused on identifying OCP subpopulations specifically induced by arthritis and the role of circulatory subset in inflammatory bone loss. As standard treatments for osteoporosis in RA mainly include risk factor elimination and pharmacological suppression of inflammation/autoimmunity (30), we propose an additional complementary approach of targeting the circulatory OCPs and preventing their homing to the inflamed joints. We defined chemokine signaling axis that enables increased migration of mouse and human OCPs, and selected putative intervention target to be aimed at early stage in the arthritis course. Circulatory CCR2^hi^ OCPs may present a significant source of osteoclastogenic cells attracted by inflammatory environment to the affected bone. Compared to resident CCR2^lo^ bone marrow OCPs, they are less proliferative, with more mature phenotype, but significantly expanded in arthritis, exhibiting potent osteoclastogenic and bone-resorbing activity. Adoptively transferred, these progenitors, labeled by the tdTomato visual marker in a pan-myeloid LysMcre/Ai9 transgenic mouse line (31), were able to home to the periarticular bone marrow compartment of the arthritic mice. We concluded that preventive blockade of the CCR2/CCL2 axis in addition to MTX treatment, during the development of collagen-induced arthritis (CIA), decreased the number of active bone-attached osteoclasts *in vivo* and OCP differentiation potential *in vitro*, thus contributing to the reduced bone resorption.

## Materials and Methods

### Mice

All the animal experiments in this study were conducted under protocols approved by the national Ethics Committee (EP 07-2/2015, EP 182/2018). Relevant guidelines and regulations of use of laboratory animals (EU Directive 2010/63/EU for animal experiments, the National Institutes of Health Guide for the Care and Use of Laboratory animals) and ARRIVE (Animal Research: Reporting *in Vivo* Experiments) guidelines for reporting animal research (32) were followed.

Male C57Bl/6 (B6; 10 to 12 weeks old) and DBA/1 (DBA; 8 to 10 weeks old) mice were used for the CIA model. LysMcre mice [from The Jackson Laboratory (stock no. 004781)] that express Cre recombinase in myeloid lineage cells, were crossed with Ai9 reporter mice [from The Jackson Laboratory (stock no. 007909)] to generate LysMcre/Ai9 mice, heterozygous for the transgene, used as donors for adoptive transfer experiment. The Ai9 mice express red fluorescent tdTomato protein following recombination in Cre-producing cells and their progeny. Mice were maintained at the animal facility of the Croatian Institute for Brain Research, University of Zagreb School of Medicine (Zagreb, Croatia) under standard housing conditions. Experimental groups contained 8 to 15 B6 mice per group, and 3 to 4 DBA mice per group. (Group size variation was mainly caused by strain-specific difference in CIA incidence, as described later.) At experimental endpoints, mice were sacrificed and tissues were harvested for analysis.

### Patients

Patients with RA, admitted to Clinical Hospital “Sveti Duh” or Clinical Hospital Center “Sestre Milosrdnice”, were included in the study after obtaining approval from the Ethics Committee (license no. 641-01/18-02/01) and informed consent from patients. A rheumatology specialist established the diagnosis of RA based on the American College of Rheumatology (ACR) 2010 criteria (33). Patients were enrolled based on the following: age of 40 or more years; disease duration of at least 5 years after diagnosis was established by a specialist; being on therapy consisting of non-steroid anti-inflammatory drugs (NSAID), disease-modifying anti-rheumatic drugs (DMARD), and/or glucocorticoids (with no history of biological therapy); and having Disease activity score including a 28-joint count (DAS28) > 3.2.

### Arthritis Induction and Visual Scoring

We used a modification of previously described protocols (34, 35) to establish model of CIA. Chicken collagen type II (CII, Sigma-Aldrich, Saint Louis, MO, USA) was prepared as 4 mg/ml solution in 0.01 M acetic acid and emulsified 1:1 with Freund’s complete adjuvant (CFA, BD Biosciences, San Jose, CA, USA) containing 4 mg/ml of heat-killed *Mycobacterium tuberculosis* strain H37RA (BD Biosciences). We immunized mice by intradermal injection (at the base of the tail) of 100 µl of emulsion to B6 and 50 µl to DBA mice. After 21 days for B6 or 19 days for DBA, mice received the same amount of emulsion containing Freund’s incomplete adjuvant (IFA) instead of CFA. Two control groups were included: non-arthritic non-treated control mice (ctrl) and CFA control mice treated with the emulsion without CII. Mice were examined in 2-day intervals and clinical signs of arthritis were scored in each paw as previously described (36): 0 = unchanged, 1 = swelling and/or redness limited to one finger/toe, 2 = swelling and/or redness of more than one finger/toe, or slight paw swelling, 3 = moderate paw swelling and redness, 4 = severe paw swelling and redness with ankylosis; with the maximum clinical score of 16 per mouse. For model evaluation, mice with clinically detectable arthritis were used.

### Flow Cytometry and Cell Sorting

Single-cell suspensions were prepared from spleens (SPL), bone marrow of distal tibia (periarticular bone marrow, PBM) and femur (systemic bone marrow, SBM), collagenase-digested tarsometatarsal (TMT) joints and peripheral blood (PBL). Cells were labeled with commercially available directly conjugated monoclonal antibodies for phenotypization and cell sorting. For labeling, we used mixtures of antibodies to lymphoid markers (anti-B220 FITC, clone RA3-6B2; anti-CD3e FITC, clone 145-2C11; anti-NK1.1 FITC, clone PK136 for B6; anti-CD49b FITC, clone DX5 for DBA), myeloid markers (anti-CD11b PE Dazzle 594, PECy-7, APC or APC-eFluor 780, clone M1/70; anti-Ly6C APC, clone HK1.4; anti-Ly6G PerCP-eFluor710, clone 1A8; anti-CD115 biotinylated or PE-Cy7, clone AFS98; anti-F4/80 APC-eFluor780, clone BM8), stem cell markers (anti-CD34 eFluor660, clone RAM34; anti-CD16/32 PECy7, clone 93; anti-Sca1 PE, clone E13-161.7; anti-CD127 biotinylated, clone A7R34; anti-CD117/cKit APC-eFluor780, clone 2B8), panhematopoietic marker (anti-CD45 BV510, PE, APC or APC-eFluor780, clone 30-F11), chemokine receptors [anti-CCR2 PE or APC/Fire750, clone SA203G11; anti-CCR3 PE, clone 83101; anti-CCR5 PE, clone HM-CCR5(7A4); anti-CCR9 PerCP-eFluor710, clone eBioCW-1.2; anti-CXCR4 PerCP-eFluor710, clone 2B11; anti-CX3CR1 BV421, PE or APC, clone SA011F11], and streptavidin coupled to PE-CF594 or APC [all from BioLegend (San Diego, CA, USA), eBiosciences (San Diego, CA, USA), R&D Systems (Bio-Techne, Abingdon, UK), or BD Biosciences] and Mouse Lineage Mixture (Invitrogen, Thermo Fisher Scientific, Waltham, MA, USA). We used 7-aminoactinomycin D (7-AAD) (BioLegend) or 4’,6-diamidino-2-phenylindole (DAPI, Sigma) staining to exclude dead cells. Stained cells were analyzed on Attune (Life Technologies, ABI, Carlsbad, CA, USA) and BD FACSAria II (BD Biosciences) instruments, and the data were analyzed using FlowJo software (TreeStar, Ashland, OR, USA).

Fluorescence-activated cell sorting (FACS) of OCPs from bone marrow (CD45^+^CD3^-^B220^-^NK1.1^-^Ly6G^-^CD11b^-/lo^CD115^+^) and spleen (CD45^+^CD3^-^B220^-^NK1.1^-^Ly6G^-^CD11b^+^CD115^+^) or their subsets was performed on the BD FACSAria II instrument, using gating strategies as described previously (5, 6). Cells were collected in tubes containing α-minimum essential medium (α-MEM)/10% fetal bovine serum (FBS, Gibco, Thermo Fisher Scientific) and further used for migration tests and cell culture. Sorting purity, verified by reanalyzing sorted cells, was greater than 99% for all experiments.

### Osteoclastogenic Cultures, Resorption, and Proliferation Assay

Sorted OCPs from SPL and PBM were plated into 96-well plates at a density of 14,000 cells/well and 7,000–28,000 cells/well, respectively, depending on the experiment. Cells were cultured in 0.2 ml/well of α-MEM/10% FBS supplemented with 30 ng/ml recombinant mouse macrophage colony-stimulating factor (rmM-CSF) (R&D Systems) and 30 ng/ml rm receptor activator of nuclear factor κB ligand (RANKL) (R&D Systems). Time lapse light microscopy was performed with EVOS FL auto imaging system (Thermo Fisher Scientific).

At the culture endpoint (3–5 days for PBM, 5–7 days for SPL), cells were fixed with 4% paraformaldehyde in PBS and stained for tartrate-resistant acid phosphatase (TRAP) expression (Leukocyte acid phosphatase kit; Sigma-Aldrich) according to the manufacturer’s instructions. Wells were scanned using Axiovert 200 light microscope (Carl Zeiss Microscopy, Jena, Germany) at 100× magnification connected to CCD camera and ZEISS ZEN 3.3 lite software (Panorama imaging). TRAP^+^ multinucleated cells with more than three nuclei were counted per well using CellProfiler 3.0 software (37) set to count cells of diameter greater than 35 pixel units in SPL and 50 pixel units in PBM as osteoclasts. Bone resorption assays were performed by seeding sorted PBM OCPs on square-shaped bovine cortical bone slices 4.4 × 4.4 × 0.2 mm (15,000 cells/slice) in osteoclastogenic culture as already described (13). After 20–25 days of culturing, slices were sonicated in 0.25 M NH_4_OH for 5 min and stained with 1% toluidine blue in 1% borax buffer for 2 min, to visualize resorption pits at 200× magnification using Axiovert 200 light microscope.

For *in vitro* proliferation assay, sorted OCPs were stained with 5- (and-6)-carboxyfluorescein diacetate succinimidyl ester (CFSE, Thermo Fisher Scientific). Briefly, cells were incubated with 5 μM CFSE in PBS/1% FBS for 8 min at room temperature and the labeling was stopped by adding an equal volume of FBS and incubated 15 min at 37°C. Cells were washed in PBS/5% FBS and seeded to 48-well plates (30,000 cells/well) in osteoclastogenic culture as already described. After 48 h of culturing, cells were collected using TrypLE Express enzyme (Gibco, Thermo Fisher Scientific), stained with 7AAD, and analyzed by flow cytometry (Attune, Life Technologies) using FlowJo (TreeStar) software and “FlowJo Proliferation function” to analyze number of cell divisions.

### *In Vitro* Migration Assay

Migration potential of sorted mouse and human OCPs was assessed using 8.0 μm pore size 24-well Transwell cell culture plate inserts (Costar, Corning Inc., Corning, NY, USA). Mouse OCPs were isolated as described above. Human peripheral blood mononuclear cells were isolated from PBL by gradient-separation (20 min at 600 g) with Histopaque (Sigma-Aldrich), labeled using the lymphoid lineage markers (anti-CD3 FITC, clone OKT3, for T lymphocytes; anti-CD19 FITC, clone HIB19, for B lymphocytes; anti-CD56 FITC, clone HCD56, for NK cells) and myeloid lineage markers (anti-CD11b APC, clone M1/70; anti-CD14 PE-Cy7, clone HCD14) and sorted using BD FACSAria II (BD Biosciences). OCPs were defined as a subset of the monocytic subpopulation bearing the CD45^+^CD3^-^CD19^-^CD56^-^CD11b^+^CD14^+^ phenotype (38).

Cells at a concentration of 2×10^4^/100 μl α-MEM/10% FBS medium were seeded into the inserts that were placed in wells containing 500 μl of medium with chemokine gradient: 10 ng/ml CCL2 (PeproTech, Rocky Hill, NJ, USA) or 1 µg/ml CX3CL1 (Novus Biologicals, Abingdon, UK) for mouse cells; 40 ng/ml CCL2 (PeproTech) or 40 ng/ml CXCL10 (PeproTech) for human cells. In a set of experiments, CCR2 or CXCR3 was blocked by adding small-molecule CCR2 receptor antagonist/small-molecule inhibitor (SMI, RS 504393, Tocris, Biotechne) or CXCR3 receptor antagonist (SMI, NBI 74330, Tocris), respectively. After 2 h of incubation at 37°C with 5% CO_2_, the upper surface of the insert membrane was washed with PBS, and the remaining cells were wiped with a cotton swab. The cells that migrated to the bottom side of the inserts were fixed with 4% paraformaldehyde, stained with DAPI, and counted (7 fields per insert) at 100× magnification using a fluorescent microscope (Axiovert 200, Carl Zeiss) and ZEISS ZEN 3.3 lite (Carl Zeiss) software. In addition, cells released in the corresponding culture well were either counted using FACS or continued to differentiate in osteoclastogenic cultures.

### Micro-Computerized Tomography Image Acquisition and Analysis

Right hind paws with distal part of tibia (skin removed) were fixed for 24 h at 4°C in 4% paraformaldehyde and moved to 70% ethanol for scanning. We used micro-computerized tomography (μCT) scanner (1076 SkyScan, SkyScan, Kontich, Belgium) at 50 kV and 200 mA, through 180° camera rotation and 0.3° rotation step, with a 0.5-mm aluminum filter, using a detection pixel size of 9 μm. Acquired scans were reconstructed with the SkyScan Recon software and analyzed using the SkyScan CTAnalyzer v.1.13. and CTVol. Bone three-dimensional analysis was performed manually delineating a fragment of talus in the horizontal plane, 0.3–0.4 mm thick. The ratio of total bone (cortical + trabecular) volume per total tissue (bone + marrow cavity) volume was calculated (the talar BV/TV, %).

### Histology, Histomorphometry, and Immunohistochemistry

Joint pathohistology was evaluated on sagittal plane sections of right tibiotalar joint. After µCT scanning, hind paws were demineralized in 14% ethylene-diamine tetraacetic acid in 3% formaldehyde for 10 days, washed in distilled water, dehydrated in increasing ethanol concentrations (70%, 96%, and 100%), moved to benzene for 12 min, and embedded in paraffin during 6 h. Serial sections were cut (6 µm) with a rotational microtome (Leica SM 2000 R, Leica Biosystems, Nussloch, Germany) and stained with hematoxylin and eosin (HE), Goldner-Masson trichrome (GMT), and histochemically for TRAP expression. Number of multinuclear TRAP^+^ osteoclasts adjacent to the bone surface was counted on distal tibia endosteal surfaces and trabecules using Axiovert 200 microscope (Carl Zeiss) and ZEN software under 200× magnification in two adjacent visual fields as region of interest (ROI); average count of three serial sections was used. GMT stain was used for histomorphometric analysis that was performed under an Axio Imager microscope (Carl Zeiss) using the OsteoMeasure software (Osteo-Metrics, Decatur, USA). Sections were analyzed under 100× magnification. ROI was selected by placing a 500 × 500 μm square in distal tibia ending beneath the joint cartilage. Subchondral and trabecular bone were delineated manually, and bone volume (BV/TV, %) and subchondral plate thickness as average interlabel distance (Ir.L.Th, μm) were calculated automatically by the software. Average measurement value on three serial sections was presented.

For immunohistochemistry, sections were deparaffinized and incubated in 10 mM sodium citrate (pH 6) at 65°C overnight for antigen retrieval. To inactivate endogenous peroxidases, 0.03% H_2_O_2_ in methanol was used for 30 min at room temperature. Sections were blocked by Rodent block M (Bio care Medical, Concord, CA, USA) and incubated with the primary antibody to CCL2 (rabbit polyclonal IgG, ab25124, Abcam, Cambridge, UK) at 4°C overnight. After incubating with Envision anti-rabbit horseradish peroxidase (HRP) (Dako, Glostrup, Denmark), identification of CCL2 was visualized by HRP substrate-chromogen 3,3′-diaminobenzidine (Vector laboratories Inc. Burlingham, CA, USA). Reaction was stopped with distilled water and hematoxylin was used to counterstain. Sections were covered using Hydromount (National Diagnostics, Atlanta, GA, USA).

### ELISA

Blood was collected from the retro-orbital plexus, and serum was separated by centrifugation after clot formation and stored at −20°C until analysis. CCL2 and CX3CL1 serum levels were assessed by ELISA [Mouse CCL2 SimpleStep ELISA Kit (Abcam), Mouse CX3CL1 SimpleStep ELISA Kit (Abcam)], following the manufacturer’s instructions. The reactions were visualized with substrate solution (tetramethylbenzidine) and optical density was determined within 15 min on a microplate reader (Bio-Rad Laboratories, Hercules, CA, USA) set to an excitation wavelength of 450 nm.

### Quantitative PCR Gene Expression Analysis

For quantitative PCR (qPCR), total RNA was extracted from harvested tissues or cultured cells (pooled 7–10 wells) using Trizol reagent (Applied Biosystems, Thermo Fisher Scientific). RNA quality was assessed using Bioanalyzer RNA PicoChip (Agilent Technologies, Santa Clara, CA, USA) and quantified using a Nanodrop spectrophotometer (Thermo Fisher Scientific). cDNA was then reverse transcribed using High Capacity RNA-to-cDNA Kit (Applied Biosystems). The amount of cDNA corresponding to 20 ng of reversely transcribed RNA was amplified in duplicate or triplicate by ABI Prism 7500 system (Applied Biosystems), using TaqMan Gene Expression Master mix and commercially available TaqMan Gene Expression Assays (Applied Biosystems) for mouse osteoclast differentiation genes *c-Fos* (Assay ID: Mm00487425_m1), *Rank* (Assay ID: Mm00437135_m1), and *CD115/c-fms* (Assay ID: Mm01266652_m1); chemokines *Ccl2* (Assay ID: Mm99999056_m1) and *Cx3cl1* (Assay ID: Mm00436454_m1); and chemokine receptors *Ccr2* (Assay ID: Mm04207877_m1) and *Cx3cr1* (Assay ID: Mm00438354_m1), and housekeeping gene *β-actin* (Assay ID: Mm02619580_g1). Gene expression was calculated from relative standard curve of gene expression in the calibrator sample (cDNA from mononuclear cells or osteoclast cultures) and presented normalized to the expression level of housekeeping gene (*β-actin*).

### Analysis of Local Osteoclast Activity Using Fluorescence Imaging

At day 34, mice were intravenously injected with 0.08 nmol/g CatK 680 FAST (NEV11000, PerkinElmer, Waltham, MA, USA) probe in PBS. CatK 680 FAST is a cathepsin K activatable imaging probe that produces fluorescent signal after cleavage by osteoclast released cathepsin K. Mice were sacrificed 24 h post injection and transferred to IVIS Spectrum imaging system (PerkinElmer) for two-dimensional epifluorescence imaging. For imaging of explants (hind paws cleaned from skin), the samples were placed in a dish. Manufacturer’s preset excitation and emission wavelength pairs and auto-exposure time were used (for CatK imaging). Obtained fluorescence intensities were analyzed by Living Image software 4.4.17106 (Perkin Elmer). Probe signal was distinguished from autofluorescence by “Spectral Unmixing” the image series (Living Image, PerkinElmer). Free draw type and circle type of ROI were set for plates and mice, respectively. Level of intensity of fluorescent signal is reported as total radiant efficiency [photon/s]/[μW/cm^2^] in the ROI.

### siRNA Application

For siRNA transfection, we used Neon Transfection System (Invitrogen, Thermo Fisher Scientific) and the corresponding kit (Neon Transfection System 10 µl Kit, Invitrogen), following the manufacturer’s instructions. Sorted PBM OCPs from CIA mice and PBL OCPs from RA patients were prepared as previously described, washed with PBS, and resuspended in T buffer (Invitrogen) at a final concentration of 2×10^4^ cells/μl. Before electroporation, specific siRNA was added to the resuspended cells (ON-TARGET plus Mouse Ccr2 or Cx3cr1 siRNA-SMART pool, and Human Cxcr3 siRNA-SMART pool, Dharmacon, Horizon Discovery, Cambridge, UK) or scrambled control siRNA (ON-TARGET plus Non-targeting Pool, Dharmacon). Electroporation was performed using 1,700 mV, 20 ms in single pulse for murine cells, and 1,000 mV, 40 ms in two pulses for human cells. Afterwards, cells were rapidly dispensed into a 96-well culture plate containing pre-warmed α-MEM/10% FBS medium without antibiotics. Final concentration of cells and siRNA was 5×10^4^ cells/well and 200 nM, respectively. Antibiotics (100 U/ml penicillin and streptomycin) were added after 3 h and cells were cultured at standard conditions for 24 h. For analysis, cells were detached using TrypLE Express (Gibco, Thermo Fisher Scientific) and washed with PBS. Viability was assessed by trypan blue exclusion, and cells were then stained for flow cytometric analysis, or resuspended in medium for migration assay or in TRIzol for RNA extraction.

### *In Vivo* Migration Tracking and Adoptive Transfer

To assess recirculation of monocytes through intravascular compartments of inflamed joints in CIA, we used *in vivo* intravascular staining with monoclonal antibodies (39). We gave mice 3 µg of anti-CD45 antibody coupled to PE intravenously to stain CD45 cells in circulation. After 3 min, we sacrificed them to obtain tissue samples, which were further stained for flow cytometric analysis, including anti-CD45 antibody coupled to APC. Intravascular cells stained double (*in vivo* and *ex vivo*) positive for CD45 (anti-CD45 PE and anti-CD45 APC), and tissue-resident populations stained only *ex vivo* (anti-CD45 APC). PBL sample was used as positive (over 90% cells stained double positive) and lymph node sample was used as negative control (less than 5% cells stained double positive).

For adoptive transfer of SPL OCPs, LysMcre/Ai9 mice were treated with CFA 2 weeks before sacrifice to expand the myeloid lineage. Their spleens were then harvested and sorted tdTomato^+^ spleen OCPs (CD45^+^CD3^-^B220^-^NK1.1^-^ Ly6G^-^CD11b^+^CD115^+^) were transferred intravenously to B6 CIA mice during two consecutive days (total 0.5×10^6^ cells/mouse). Host mice (PBL, SPL, and PBM) were analyzed for the presence of graft OCPs identified by tdTomato expression.

### *In Vivo* CCL2/CCR2 Axis Blockade

For *in vivo* treatment with a small-molecule CCR2 receptor antagonist (SMI, RS 504393, Tocris, Biotechne) starting at day 19 (± 2 days) after primary immunization (immediately prior to secondary immunization), we randomly allocated asymptomatic DBA mice to experimental groups (*n* = 3–4 per group per experiment). Every 48 h until the end of the experiment, mice were given intraperitoneal injections of 4 mg/kg SMI (dissolved in DMSO to 10 mM and diluted in PBS for application, SMI group) or 2 mg/kg methotrexate (MTX, Ebetrexat, Sandoz; MTX group) or both at the same dosage (SMI+MTX group). Untreated CIA (NT group) received 100 μl of sterile PBS or vehicle (DMSO in PBS, DMSO group).

### Statistical Analysis

Each experiment was performed at least three times and numbers of independent experiments performed and mice analyzed per group are indicated in figure legends. The results were statistically analyzed using MedCalc Statistical Software version 13.1.2 (MedCalc Software, Ostend, Belgium). Kolmogorov–Smirnov test was used to verify normality of data distribution. Results are presented as median with interquartile range (IQR) and data are plotted as box-and-whisker diagrams, where medians are represented by middle horizontal lines, boxes represent the IQR, whiskers represent 1.5 times the IQR, and outliers are represented by circles. For some measurements (indicated in figure legends), values were normalized to the average of the control group. Clinical scores for preventive treatment follow-up are presented as median per group per experiment (total of six experiments). Two outcomes, IVIS Spectrum post-treatment fluorescence imaging and qPCR post-treatment *c-Fos* gene expression in OCP cultures, were measured in the representative experiment and are presented as individual values. Differences between groups were analyzed by Mann–Whitney *U*-test or by the non-parametric Kruskal–Wallis test followed by Conover test for group-to-group comparisons. For results that included simultaneous testing of independent variables, correction for multiple comparisons was performed using Holm–Bonferroni method (indicated in figure legends). In all experiments, α-level was set at 0.05.

## Results

### Phenotypic Dissection of Myeloid Lineage and Osteoclastogenic Subsets in Arthritis

**Figure 1:**
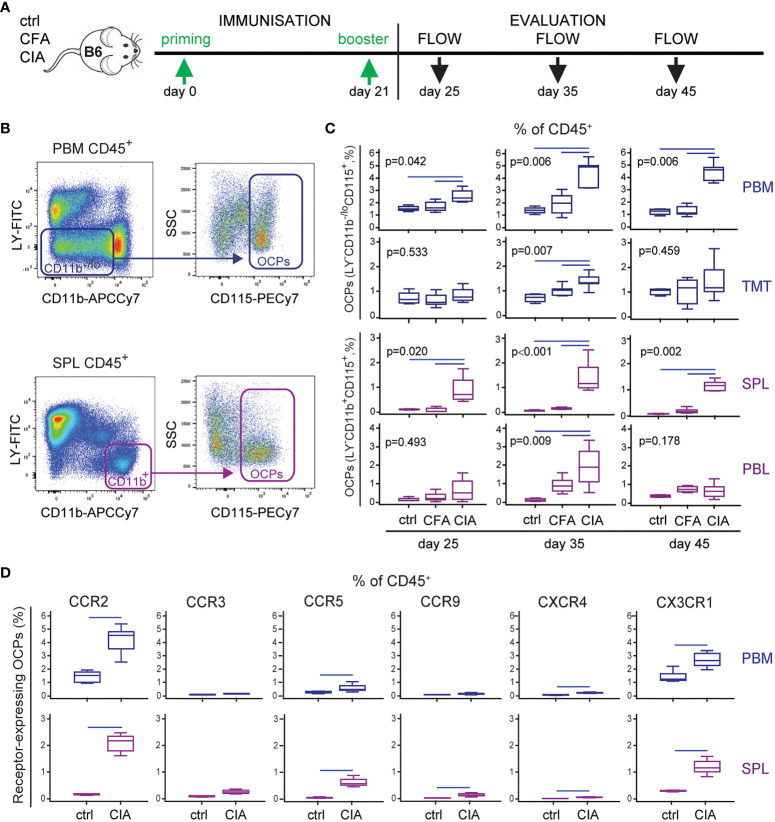
Increased frequency of osteoclast progenitors (OCPs) in B6 mice with collagen-induced arthritis (CIA). **(A)** Experimental timeline for CIA model in B6 mice. To induce arthritis, mice were immunized with chicken collagen type II emulsified with complete Freund’s adjuvant (CFA) at day 0, followed by a booster dose at day 21 (CIA group). Likewise, CFA control group received only adjuvant, and control (ctrl) group was left untreated. Three time points during arthritis development were selected for analysis. **(B)** Gating strategy for flow cytometric analysis of OCPs in medullar compartment [distal tibia bone marrow (periarticular, PBM) and tarsometatarsal joints (TMT)], bearing the phenotype CD45^+^CD3^-^B220^-^NK1.1^-^CD11b^-/lo^CD115^+^, and peripheral compartment [spleen (SPL) and peripheral blood (PBL)], bearing the phenotype CD45^+^CD3^-^B220^-^NK1.1^-^CD11b^+^CD115^+^. Representative dot plots for PBM and SPL are shown. **(C)** Frequency of OCPs at different time points (25, 35, and 45 days after primary immunization) in analyzed tissues of ctrl, CFA, and CIA mice, determined using the previously described gating strategy. The OCP percentage of CD45^+^ population is presented. **(D)** Immunophenotyping of chemokine receptors (CCR2, CCR3, CCR5, CCR9, CXCR4, and CX3CR1) on PBM and SPL OCPs (35 days after primary immunization). The percentage of chemokine receptor-expressing OCPs of CD45^+^ population is presented. The experiments were repeated three times and pooled data are shown (*n* = 6–10 mice per group). Values are presented as medians (middle horizontal lines), boxes represent the interquartile range (IQR), whiskers represent 1.5 times the IQR. Statistically significant difference was determined at *p* < 0.05, Mann–Whitney *U*-test **(D)**, or Kruskal–Wallis test (*p*-value marked on the graph) with Conover *post-hoc* test for group-to-group comparisons **(C)**; correction for multiple comparisons **(D)** was performed using Holm–Bonferroni method; line denotes significant difference between groups. LY—CD3/B220/NK1.1.

In line with the previous studies of other authors and our group (6, 36, 40), we confirmed myeloid lineage expansion in arthritic mice. In particular, we profiled dynamic changes of OCP subsets with the progression of arthritis (**Figure 1A**). The frequency of OCPs, identified as CD3^-^B220^-^NK1.1^-^CD11b^-/lo^CD115^+^ cells in periarticular tissues (distal tibia PBM and cells released from TMT joints) and CD3^-^B220^-^NK1.1^-^CD11b^+^CD115^+^ cells in peripheral tissues (SPL and PBL) (**Figure 1B**), was increased among hematopoietic CD45^+^ cells in arthritis (**Figure 1C**). These subsets reach the maximum expansion at day 35 post primary immunization (**Figure 1C**), in parallel with the fully developed clinical arthritis (see **Figure 5**). Myeloid infiltrate in distal tibia of arthritic CIA mice differed significantly from non-arthritic groups: ctrl (control non-treated mice) and CFA (mice treated only with adjuvant), in both cellularity and frequency of myeloid cells, containing the OCP population (**Figure 1C**, see **Figure 5**). Comparison between the CFA group (non-specific inflammation induced by complete Freund’s adjuvant) and the CIA group (specific autoimmune response to collagen) clearly showed that OCP expansion is specifically associated with arthritis.

Besides dissecting OCP subsets, we further analyzed the proportion of committed early myeloid progenitors in the bone marrow and mature myeloid lineage progenies in the bone marrow and spleen (**Supplementary Figures 1A, B**). The results suggest that myeloid lineage is induced from the stage of common myeloid progenitors in the bone marrow, resulting in an increased frequency of mature components, including granulocytes, macrophages, and monocytes among spleen and bone marrow cells.

Based on the finding that OCP subsets are expanded in arthritis not only adjacent to the affected joints, but also in the circulation, we hypothesized that extramedullary tissues represent the reservoir of osteoclastogenic cells susceptible to inflammatory signals. Therefore, we determined the frequency of OCPs expressing chemokine receptors within the hematopoietic CD45^+^ compartment (**Figure 1D**), since chemokine signals may be responsible for their homing to the affected joints. Among a panel of chemokine receptors, only the percentage of CCR2 and CX3CR1 expressing OCPs from both PBM and SPL were substantial, whereas other receptors were expressed on a minority of OCPs (**Supplementary Figure 2**, showing representative dot plots of immediate parent population). The largest arthritis-induced increase was observed for CCR2, CCR5, and CX3CR1 expressing OCP subsets (**Figure 1D**), but due to their sufficient expression in both (ctrl and CIA) groups, we selected CCR2 and CX3CR1 for further investigation.

### CCL2/CCR2 and CX3CL1/CX3CR1 Axes in OCPs of Arthritic Mice

**Figure 2:**
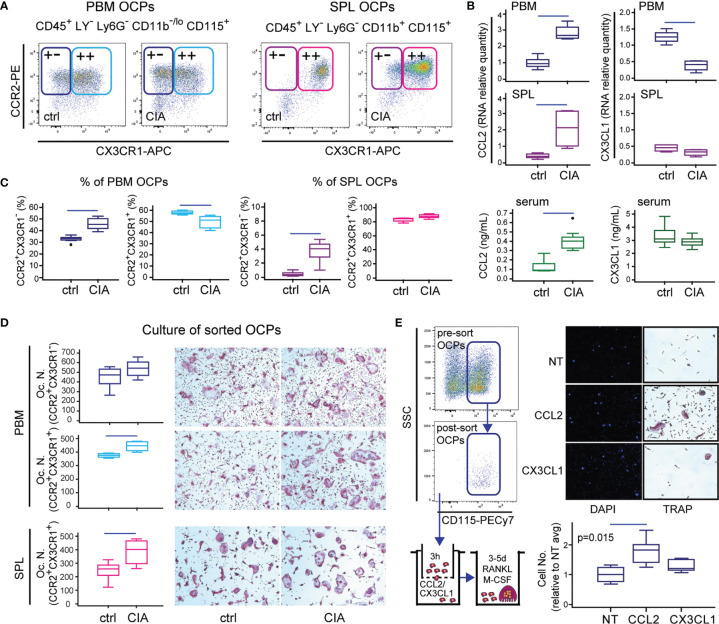
Importance of the CCL2/CCR2 and CX3CL1/CX3CR1 axes in B6 mice with collagen-induced arthritis (CIA). **(A)** Representative dot plots for flow cytometric analysis of CCR2 and CX3CR1 expression on osteoclast progenitors (OCPs) defined as CD45^+^CD3^-^B220^-^NK1.1^-^Ly6G^-^CD11b^-/lo^CD115^+^ cells in periarticular bone marrow (PBM) and CD45^+^CD3^-^B220^-^NK1.1^-^Ly6G^-^CD11b^+^CD115^+^ cells in spleen (SPL) of control (ctrl) and CIA mice. **(B)** qPCR analysis of chemokine gene expression levels in PBM and SPL presented as relative quantity of RNA, and serum levels of chemokine ligands measured by enzyme-linked immunosorbent assay (ELISA), in ctrl and CIA mice. **(C)** Proportion of single-positive (CCR2^+^CX3CR1^-^) and double-positive (CCR2^+^CX3CR1^+^) OCPs in PBM and SPL of ctrl and CIA mice. **(D)** Osteoclastogenic potential of single-positive (PBM CCR2^+^CX3CR1^-^) and double-positive (PBM and SPL CCR2^+^CX3CR1^+^) OCPs of ctrl and CIA mice. Sorted cells were plated and stimulated by receptor activator of nuclear factor κB ligand (RANKL) and macrophage colony-stimulating factor (M-CSF). Differentiated tartrate-resistant acid phosphatase (TRAP)^+^ multinucleated osteoclast-like cells were counted per well (Oc.N.). Representative microphotographs of *in vitro* differentiated TRAP^+^ osteoclast-like cells are presented (magnification 100×). **(E)** *In vitro* migration assay of sorted PBM OCPs of arthritic mice (CIA) using Transwell system. Cells migrated to the bottom membrane of the insert were stained with 4’,6-diamidino-2-phenylindole (DAPI), counted (*n* = 2 wells per group; 7 fields per well), and normalized to the average value of non-treated group (NT avg). Cells that migrated to the lower chamber were cultured with RANKL and M-CSF. Representative images of NT, CCL2- and CX3CL1-attracted cells (nuclei stained with DAPI) and *in vitro* differentiated TRAP^+^ osteoclast-like cells are presented (magnification 100×). The experiments were repeated three times and pooled data are shown (*n* = 6–10 mice per group). Values are presented as medians (middle horizontal lines), boxes represent the interquartile range (IQR), whiskers represent 1.5 times the IQR, and outliers are represented by circles. Statistically significant difference was determined at *p* < 0.05, Mann–Whitney *U*-test **(B–D)** or Kruskal–Wallis test (*p*-value marked on the graph) with Conover *post-hoc* test for group-to-group comparisons **(E)**; correction for multiple comparisons **(B)** was performed using Holm–Bonferroni method; line denotes significant difference between groups. LY—CD3/B220/NK1.1.

Recent literature strongly evidenced that circulating cells are a source of OCPs, especially in conditions that perturb the integrity of the bone marrow microenvironment (9, 12). Therefore, we further dissected peripheral and periarticular OCP subsets among the myeloid non-granulocyte (Ly6G^-^) population, and assessed the expression of CCR2 and CX3CR1 receptors that potentially mediate chemoattraction (**Figure 2A**). Exclusion of Ly6G^+^ granulocytes that cannot generate osteoclasts proved to be essential for the precise determination of OCP frequency. Expression of CD115 is indispensable for the ability of osteoclast generation, since the CD115^-^ subset does not generate osteoclasts (not shown). Majority of SPL OCPs co-express CCR2 and CX3CR1 alongside CD115, whereas a negligible proportion has a low expression of these chemokine receptors. In PBM, CCR2 was abundantly expressed on CD115^+^ OCPs, whereas CX3CR1 was less frequent (approximately 2/3 double-positive cells) (**Figure 2A**).

We then analyzed the expression of corresponding ligands and found a decrease of CX3CL1 expression particularly in PBM and TMT, while the expression of CCL2 was significantly increased in both medullary and extramedullary tissues of mice with arthritis in comparison to control (**Figure 2B** and **Supplementary Figure 3**). Accordingly, the serum level of CCL2 was significantly higher in arthritic mice, whereas the level of CX3CL1 was comparable to control (**Figure 2B**).

To further assess the comparative importance of CCR2 versus CX3CR1 expression for osteoclastogenic potential, we determined the relative frequency of CCR2^+^CX3CR1^-^ and CCR2^+^CX3CR1^+^ subsets in PBM and SPL of control and arthritic mice, and sorted these subpopulations for osteoclastogenic cultures (**Figures 2C, D**). The results showed that in PBM, only CCR2^+^CX3CR1^-^ subpopulation is enlarged in arthritic mice, whereas CCR2^+^CX3CR1^+^ subpopulation was proportionally downregulated (**Figure 2C**, left). In SPL of control mice, virtually all OCPs were double positive, with the increase in CCR2^+^CX3CR1^-^ cell frequency in arthritis (**Figure 2C**, right). Furthermore, both subpopulations were efficient in generating TRAP^+^ multinucleated osteoclasts in culture, especially in arthritic mice (**Figure 2D**). The highest number of osteoclasts was detected in cultures of CCR2^+^CX3CR1^-^ PBM OCPs, indicating that CX3CR1 expression is not essential for osteoclastogenic potential. Regarding the functional difference between bone marrow and peripheral OCPs, it is important to note that peripheral OCPs need to be seeded at a higher density and a longer duration for efficient osteoclast generation.

Since a substantial proportion of OCPs expressed CCR2 and CX3CR1 chemokine receptors, we expected that they can respond to the CCL2 and CX3CL1 chemokine gradient and functionally tested their migratory potential using the Transwell culture system (**Figure 2E**). Sorted OCPs from the periarticular compartment, exposed to chemotactic gradient, showed significantly increased chemotaxis toward CCL2, whereas CX3CL1 did not enhance progenitor migration. Enhanced migration was shown as an increased number of cells attached to the outer side of the Transwell membrane as well as an increase in the total number of cells released outside the Transwell chamber. Moreover, cells that migrated through the Transwell membrane in the corresponding culture well generated multinuclear osteoclasts only in the CCL2-suplemented group (**Figure 2E**).

### Level of CCR2 Expression Defines Functionally and Morphologically Distinct OCP Subsets

**Figure 3:**
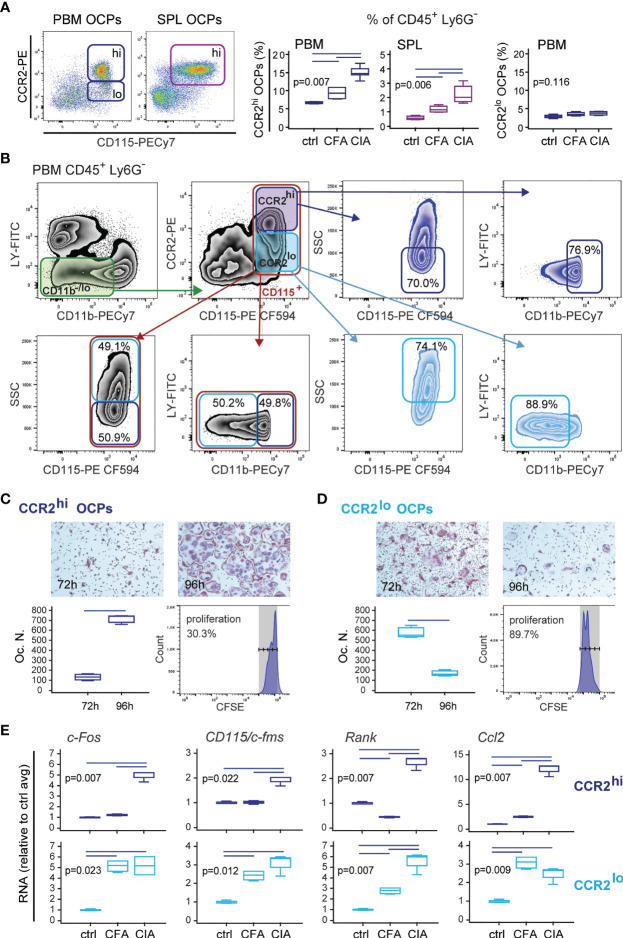
Properties of CD45^+^CD3^-^B220^-^NK1.1^-^Ly6G^-^CD11b^-/lo^CD115^+^CCR2^hi^ (CCR2^hi^) and CD45^+^CD3^-^B220^-^NK1.1^-^Ly6G^-^CD11b^-/lo^CD115^+^CCR2^lo^ (CCR2^lo^) osteoclast progenitor (OCP) subpopulations in B6 mice with collagen-induced arthritis (CIA). **(A)** Frequency of CCR2^hi^ and CCR2^lo^ OCPs in periarticular bone marrow (PBM) and spleen (SPL) in control (ctrl), complete Freund’s adjuvant treated mice (CFA), and arthritic mice (CIA). Representative dot plots are shown. **(B)** Representative flow cytometry data of the properties of CCR2^hi^ and CCR2^lo^ OCPs in arthritic PBM. Greater proportion of CCR2^hi^ cells is of lower SSC signal and CD11b low positive. Greater proportion of CCR2^lo^ cells shows higher SSC signal than CCR2^hi^ cells and is CD11b negative. Red gate—total CD115^+^ cells; dark blue gate—CCR2^hi^ subset; light blue gate—CCR2^lo^ subset. **(C, D)** Proliferative and osteoclastogenic potential of CCR2^hi^ **(C)** and CCR2^lo^ **(D)** subpopulations. Sorted cells were stimulated for 72 h and 96 h by receptor activator of nuclear factor κB ligand (RANKL) and macrophage colony-stimulating factor (M-CSF) in culture plates at the same density (7,000 cells/well). Representative microphotographs of *in vitro* differentiated tartrate-resistant acid phosphatase (TRAP)^+^ osteoclast-like cells are presented (magnification 100×). Differentiated TRAP^+^ multinucleated osteoclast-like cells were counted per well (Oc.N.). For proliferation assay, sorted cells were stained with carboxyfluorescein diacetate succinimidyl ester (CFSE) before culturing for 2 days with RANKL and M-CSF, and flow cytometric analysis. **(E)** qPCR analysis of *c-Fos, CD115/cFms, Rank*, and *Ccl2* gene expression levels in sorted CCR2^hi^ and CCR2^lo^ progenitor subpopulations after culturing with RANKL and M-CSF for 2 days, presented as relative quantity of RNA in ctrl, CFA, and CIA mice, and normalized to the average value of control group (ctrl avg). The experiments were repeated three times and pooled data are shown (*n* = 6–10 mice per group). Values are presented as medians (middle horizontal lines), boxes represent the interquartile range (IQR), whiskers represent 1.5 times the IQR. Statistically significant difference was determined at *p* < 0.05, Mann–Whitney *U*-test **(C, D)**, Kruskal–Wallis test (*p*-value marked on the graph) with Conover *post-hoc* test for group-to-group comparisons **(A, E)**; correction for multiple comparisons **(E)** was performed using Holm–Bonferroni method; line denotes significant difference between groups. *CD115/cFms*—colony stimulating factor 1 receptor (*Csf1r*), *Rank*—receptor activator of nuclear factor κB, LY—CD3/B220/NK1.1.

Based on the above findings, we focused on the CCL2/CCR2 axis as the important chemotactic signal contributing to enhanced homing and differentiation of OCPs in the periarticular tissue. According to CCR2 expression, OCP populations can be further divided into CCR2^lo^ and CCR2^hi^ subsets, of which only the latter is significantly induced in both SPL and PBM of arthritic mice compared to control and adjuvant-treated mice (**Figure 3A**). Both subsets possess osteoclastogenic and bone-resorbing activity, but differ in phenotype, morphology, and proliferation potential (**Figures 3B–D** and **Supplementary Figure 4**). CCR2^lo^ subset, present only in PBM, is higher in SSC (higher complexity) and lower in CD11b expression (mostly CD11b^-^) (**Figure 3B**). These cells rapidly generate numerous TRAP^+^ bone-resorbing osteoclasts if seeded at lower density, due to their higher proliferative potential (**Figure 3D** and **Supplementary Figure 4**). Longer culture is associated with the decline in the number of TRAP^+^ osteoclasts, corresponding to apoptosis of rapidly matured multinucleated cells. On the other hand, the CCR2^hi^ subset corresponds to small, non-granular cells within CD11b^lo^ gate (**Figure 3B**), which divide slowly and require either higher density or longer cultures for maximum osteoclast yield (**Figure 3C** and **Supplementary Figure 4**). Based on the presented phenotype and functional characterization as well as distribution between medullary and extramedullary tissues, it can be proposed that the CCR2^hi^ subset, present in both PBM and SPL, represents a distinct peripheral OCP subpopulation, which can be attracted through circulation to the sites of bone resorption.

Finally, we analyzed the expression of osteoclast-differentiation genes in CCR2^hi^ and CCR2^lo^ OCP subsets at day 2 of osteoclastogenic (M-CSF- and RANKL-stimulated) culture. Expression of osteoclast-differentiation genes was specifically associated with arthritis only in CCR2^hi^ OCPs, whereas expression in CCR2^lo^ subset was induced with both adjuvant (CFA) and immunization (CIA) (**Figure 3E**). The relative quantity of *c-Fos* was similar in both subsets, whereas *CD115/c-Fms* and *Rank* were lower in the CCR2^hi^ subset, possibly corresponding to their slower proliferation and differentiation (**Figures 3C, D**). Interestingly, the CCR2^hi^ subset of OCPs from arthritic mice potently expressed CCL2, suggesting an autocrine action.

### Interference With CCL2/CCR2 Signal *In Vitro*

**Figure 4:**
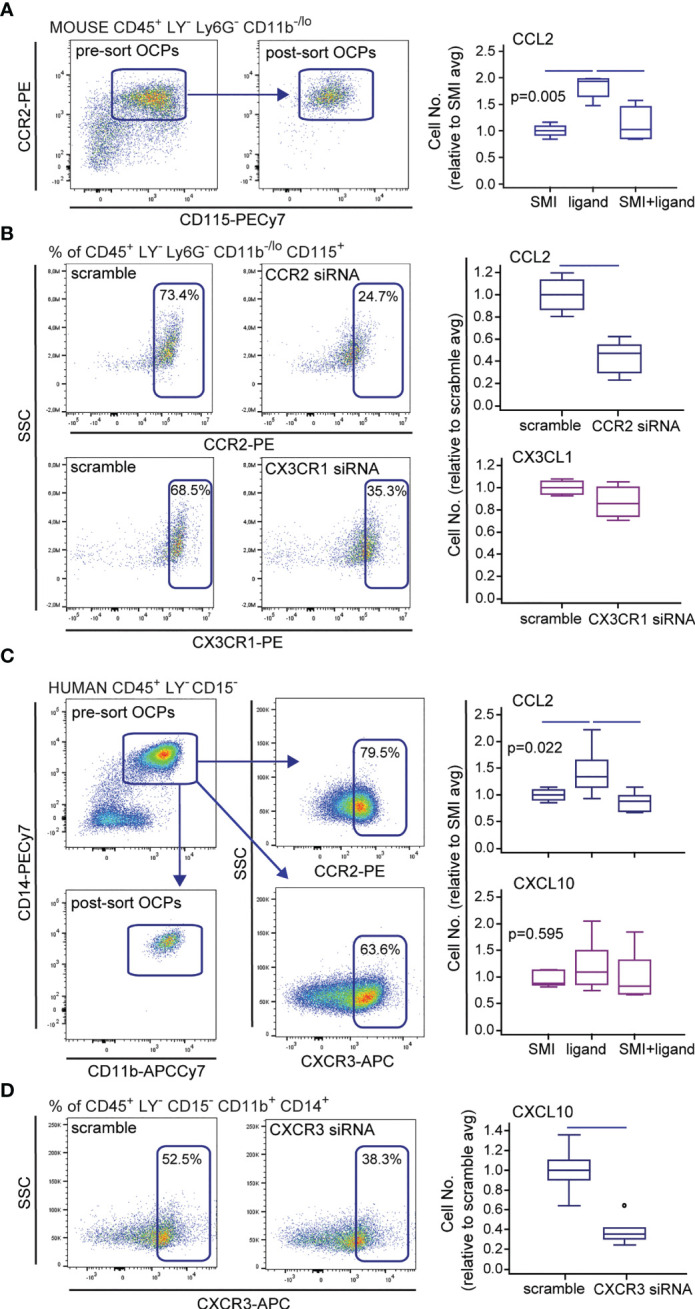
Effect of small-molecule inhibitors (SMIs) and small interfering (si)RNA on migration of mouse and human osteoclast progenitors (OCPs) *in vitro*. Representative flow cytometry data of sorted population and chemokine receptor expression, and siRNA knockdown (CCR2 and CX3CR1 for mouse and CXCR3 for human cells) are shown on dot plots. **(A)** Sorted mouse periarticular bone marrow (PBM) OCPs bearing the phenotype CD45^+^CD3^-^B220^-^NK1.1^-^Ly6G^-^CD11b^-/lo^CD115^+^ from arthritic mice (CIA) were subjected to the *in vitro* migration assay toward the CCL2 gradient under the CCR2 SMI treatment, using the Transwell system. **(B)** Sorted mouse PBM OCPs bearing the phenotype CD45^+^CD3^-^B220^-^NK1.1^-^Ly6G^-^CD11b^-/lo^CD115^+^ from arthritic mice (CIA) were subjected to the *in vitro* migration assay toward the CCL2 and CX3CL1 gradient following CCR2 or CX3CR1 siRNA treatment, respectively, using the Transwell system. **(C)** Sorted human peripheral blood (PBL) OCPs bearing the phenotype CD45^+^CD3^-^CD19^-^CD56^-^CD11b^+^CD14^+^ from arthritic patients (RA) were subjected to the *in vitro* migration assay toward the CCL2 or CXCL10 gradient under the CCR2 or CXCR3 SMI treatment, respectively, using the Transwell system. **(D)** Sorted human PBL OCPs bearing the phenotype CD45^+^CD3^-^CD19^-^CD56^-^CD11b^+^CD14^+^ from RA patients were subjected to the *in vitro* migration assay toward the CXCL10 gradient following CXCR3 siRNA treatment, using the Transwell system. Cells migrated to the bottom membrane of the insert were stained with 4’,6-diamidino-2-phenylindole (DAPI) and counted. Values are normalized to the average value of control group (SMI or scramble avg), and presented as medians (middle horizontal lines), boxes represent the interquartile range (IQR), whiskers represent 1.5 times the IQR, and outliers are represented by circles. The experiments were repeated three times and pooled data are shown (*n* = 6–8 Transwells per group). Statistically significant difference was determined at *p* < 0.05, Kruskal–Wallis test (*p*-value marked on the graph) with Conover *post-hoc* test for group-to-group comparisons **(A, C)**, Mann–Whitney *U*-test **(B, D)**; line denotes significant difference between groups. LY—CD3/B220/NK1.1 for mouse cells, CD3/CD19/CD56 for human cells.

So far, results suggest the role of CCL2 chemotaxis in homing and increased activity of OCPs in arthritis. Therefore, we tested the effect of CCR2/CCL2 axis disruption on *in vitro* migration of sorted OCPs, using two models—CCR2 SMI treatment and siRNA knockdown in the Transwell culture system (**Figure 4**). For additional comparison, CX3CR1 knockdown was also used to block the CX3CR1/CX3CL1 axis and assess the effect on OCP migration. Both the CCR2 SMI and siRNA model successfully suppressed the migration of OCPs (**Figures 4A, B**), whereas CX3CR1 knockdown did not have a significant effect (**Figure 4B**). CCR2 knockdown by siRNA was confirmed by flow cytometry (**Figure 4B**) and at gene expression level (not shown). Although both models significantly interfered with CCL2/CCR2 signal, siRNA transfection by electroporation was associated with severe loss of viable cells (<30% cell viability), possibly due to additional cell stress by prior exposure to FACS and overall fragility of primary monocytes to electroporation (41).

Since we previously showed that a substantial proportion of human peripheral blood OCPs also express chemokine receptors, particularly CCR2 and CXCR3 (13), we applied a similar experimental design to verify the role of corresponding ligands CCL2 and CXCL10, respectively, in human OCP migration. CCR2 SMI significantly suppressed OCP chemotaxis, whereas CXCR3 SMI was ineffective (**Figure 4C**). Although CXCR3 siRNA knockdown resulted in reduced migration (**Figure 4D**), transfection procedure reduced cell viability similarly as in mouse cells (not shown). Our study is the first to show that circulatory OCPs are highly susceptible to CCL2 gradient, which warrants revisiting the CCR2 blockade as a promising therapeutic strategy.

### Homing of CCR2-Positive Circulatory OCPs to the Periarticular Compartment

**Figure 5:**
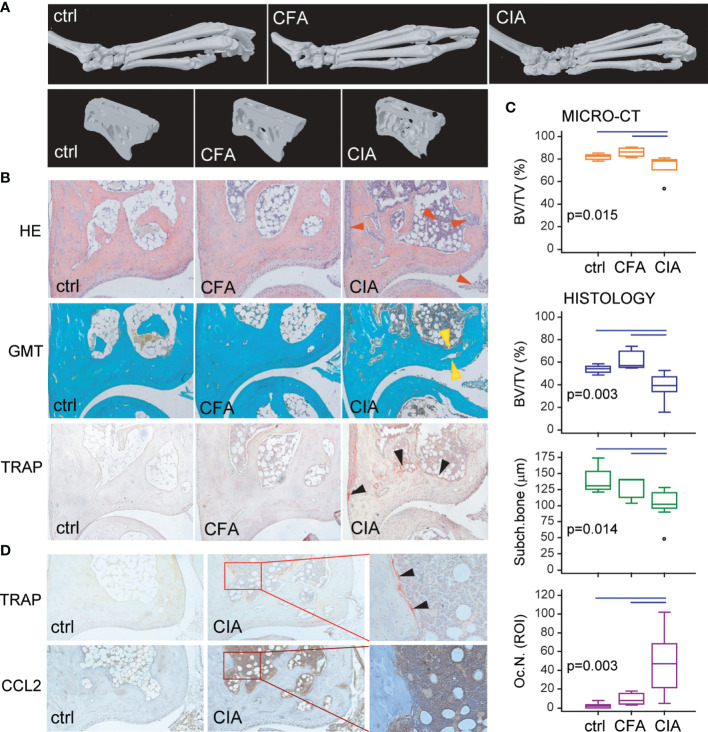
Arthritis-induced bone destruction evaluated by micro-computerized tomography (μCT) and histopathology of control (ctrl), Complete Freund’s Adjuvant treated control (CFA) and arthritic B6 mice (CIA). **(A)** Representative three-dimensional reconstructions of hind paws and fragments of talus scanned by μCT. **(B)** Representative images of tibiotalar joint histology sections in sagittal plane stained with hematoxylin and eosin (HE), Goldner-Masson trichrome (GMT), and histochemically stained for tartrate-resistant acid phosphatase (TRAP) (magnification 100×); red arrows—areas of infiltration and pannus invasion; yellow arrows—subchondral bone plate; black arrows—areas with TRAP^+^ osteoclasts. **(C)** Talar bone volume analysis by μCT [total bone volume/total tissue volume, BV/TV (%)], histomorphometric analysis of distal tibia [subchondral and trabecular bone/total tissue volume, BV/TV (%)], subchondral plate thickness [subch. bone interlabel distance (µm)], and number of TRAP^+^ osteoclasts (Oc.N.) at endosteal surfaces of distal tibia (region of interest, ROI) of ctrl, CFA, and CIA mice. The experiments were repeated three times and data from a representative experiment are shown (*n* = 6–10 mice per group). Values are presented as medians (middle horizontal lines), boxes represent the interquartile range (IQR), whiskers represent 1.5 times the IQR, and outliers are represented by circles. Statistically significant difference was determined at *p* < 0.05, Kruskal–Wallis test (*p*-value marked on the graph) with Conover *post-hoc* test for group-to-group comparisons; line denotes significant difference between groups. **(D)** Representative images of tibiotalar joint sections histochemically stained for TRAP activity and immunohistochemically stained for CCL2 expression in ctrl and CIA mice (magnification 100×); selected area (in rectangle, magnification 200×); black arrows—areas with TRAP^+^ osteoclasts.

The biological importance of the *ex vivo* characterized migratory OCP subset specifically induced by arthritis was further validated *in vivo*. We first confirmed inflammation-induced bone loss in B6 arthritic mice, with hind paw bone erosions and joint destruction, as shown by µCT and histology (**Figures 5A, B**). The proportion of total bone (cortical + trabecular) per tissue volume was significantly lower in mice with CIA compared to ctrl and CFA groups (**Figure 5C**). Analysis of tibiotalar joint histology showed a flared osteitis in mice with arthritis, with extensive infiltrate, subchondral plate thinning, cartilage damage, and marginal pannus invasion (**Figures 5B, C**). A significantly increased number of TRAP^+^ osteoclasts was observed at sites of bone destruction compared to control mice and adjuvant-treated mice (**Figures 5B, C**). Immunohistochemical staining for CCL2 protein demonstrated increased expression in the affected joints of arthritic mice compared to controls, especially in synovia, cartilage, and bone marrow (**Figure 5D**). Strong CCL2 staining in the bone marrow compartment, containing hematopoietic infiltrate, was juxtaposed to TRAP^+^ osteoclasts covering the bone surface.

**Figure 6:**
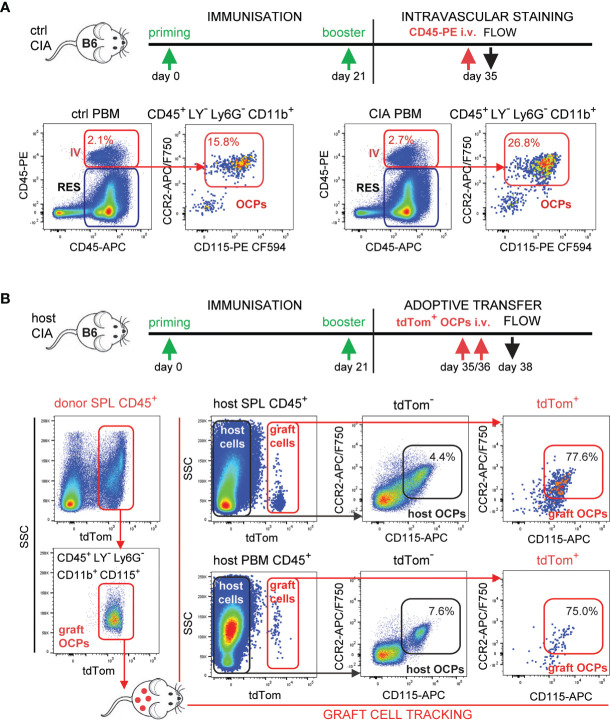
Tracking of the migration and homing of circulatory osteoclast progenitors (OCPs) to the affected sites in B6 mice with collagen-induced arthritis (CIA). **(A)** Experimental timeline for CIA model and intravascular (IV) staining in B6 mice. Increased recirculation of OCPs (CD45^+^CD3^–^B220^-^NK1.1^-^Ly6G^-^CD11b^+^CD115^+^) from arthritic mice (CIA) in comparison to control (ctrl). Hematopoietic blood cells were stained *in vivo* by intravenous (i.v.) anti-CD45 fluorescent (PE) antibody. Mice were sacrificed and harvested periarticular bone marrow (PBM) was stained *ex vivo* by anti-CD45 fluorescent (APC) antibody. Double-positive cells represent the intravascular compartment (PE^+^APC^+^ IV), whereas single-positive cells represent the resident compartment (PE^–^APC^+^ RES) within PBM. Representative dot plots and cell frequencies for ctrl and CIA are shown. **(B)** Experimental timeline for CIA model and adoptive transfer in B6 mice. Adoptive transfer of sorted tdTomato-positive spleen (SPL) OCPs (CD45^+^CD3^-^B220^-^NK1.1^-^Ly6G^-^CD11b^+^CD115^+^) from LysMcre/Ai9 mice to the host B6 mice with arthritis was performed (total 0.5×10^6^ cells/mouse i.v.). Representative dot plots of sorting efficiency for tdTomato^+^ graft OCPs are shown on the left. Frequencies of host (tdTomato^-^) and graft (tdTomato^+^) OCPs (CD45^+^CD115^+^CCR2^+^) in SPL and PBM are presented on the right. LY—CD3/B220/NK1.1.

To further address the hypothesis that circulatory CCR2^hi^ OCPs increasingly home to the inflamed joints contributing to bone resorption, we applied *in vivo* intravascular staining with anti-CD45 monoclonal antibodies as a pan-hematopoietic marker (**Figure 6A**). Intravascular compartment of the periarticular region contained similar proportion (approximately 2%) of hematopoietic cells in control and arthritic mice, detected as double-positive (*in vivo* PE-anti-CD45 end *ex vivo* APC-anti-CD45 labeled) subset as opposed to single-positive (*ex vivo* APC-anti-CD45 labeled) resident subset. Importantly, the proportion of OCPs among intravascular (double labeled, red arrows) CD45^+^ cells was almost doubled in arthritic compared to control mice (**Figure 6A**), indicating increased attraction.

Finally, we directly confirmed the homing of circulatory OCPs by adoptive transfer of myeloid cells labeled by tdTomato fluorescent protein as visual marker in LysMcre/Ai9 transgenic mice. LysMcre mouse line expresses Cre recombinase in myeloid lineage cells, including OCPs (31). We proved that the defined subpopulation of spleen OCPs (CD45^+^CD3^-^B220^-^NK1.1^-^Ly6G^-^CD11b^+^CD115^+^) generates TRAP^+^ osteoclasts expressing tdTomato *in vitro* (**Supplementary Figure 5A**). The same population of tdTomato^+^ SPL OCPs was FACS sorted and adoptively transferred to host B6 wild-type mice at the time of arthritis development (total 0.5×10^6^ cells/mouse at days 35 and 36 post-primary immunization) by retro-orbital i.v. injection (**Figure 6B**, left dot plots). Mice were sacrificed at day 38 post-primary immunization and analyzed for grafted tdTomato^+^ OCPs. The target population was detected in SPL and in PBM (**Figure 6B**, by flow cytometry, **Supplementary Figure 5B** by histology), as well as in peripheral blood (not shown) of the host mice. Parallel analysis of corresponding host and graft OCP populations (CD45^+^CD115^+^CCR2^+^tdTomato^-^ and CD45^+^CD115^+^CCR2^+^tdTomato^+^, respectively), showed 10-fold higher frequency of OCPs among graft compared to host cells for both SPL and PBM compartments (**Figure 6B**, right dot plots).

### *In Vivo* Course of CIA Under Preventive CCL2/CCR2 Signal Blocking in Arthritic DBA Mice

**Figure 7:**
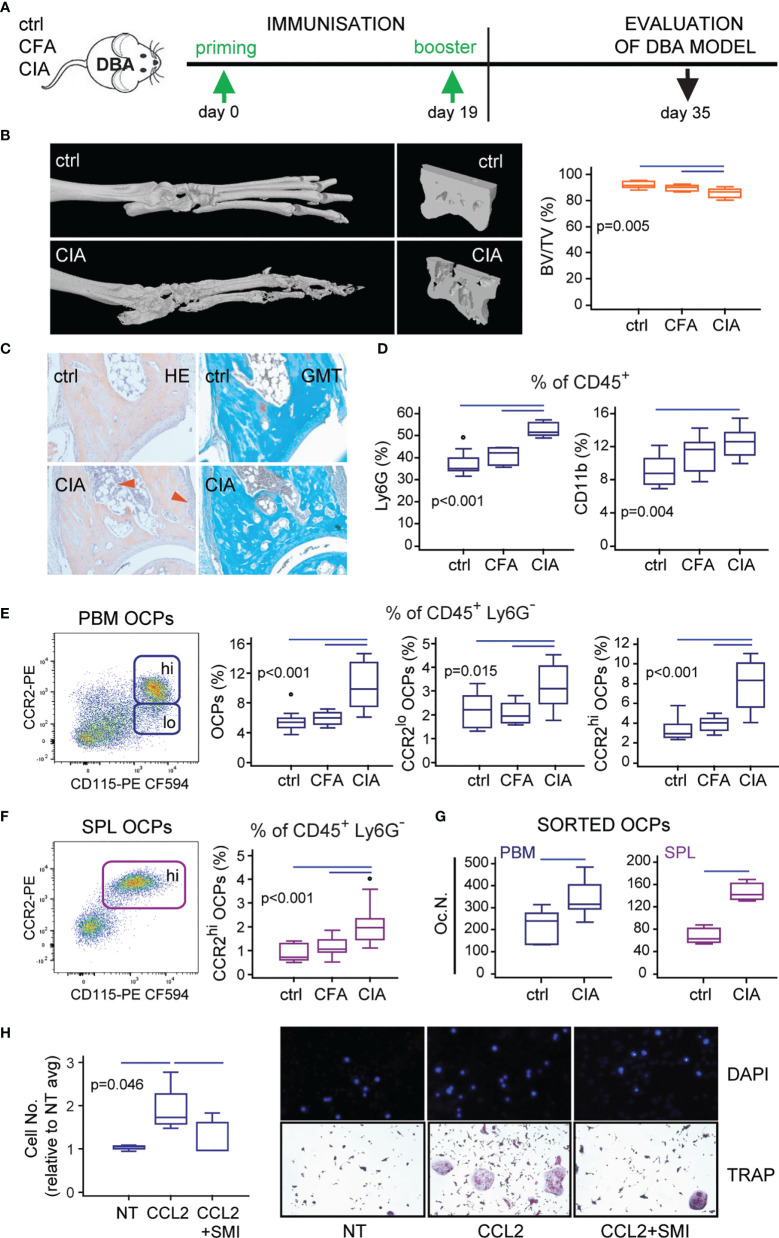
Confirmation of the corresponding model of collagen-induced arthritis (CIA) in DBA mice. **(A)** Experimental timeline for CIA model in DBA mice. Mice were immunized with chicken collagen type II emulsified with complete Freund’s adjuvant (CFA) at day 0, followed by a booster dose at day 19 (CIA group). **(B)** Representative three-dimensional reconstructions of hind paws and fragments of talus scanned by micro-computed tomography (μCT) with talar bone volume analysis [total bone volume/total tissue volume, BV/TV (%)] of control (ctrl), complete Freund’s adjuvant treated control (CFA), and arthritic mice (CIA). **(C)** Images of tibiotalar joint sagittal plane sections stained with hematoxylin and eosin (HE) and Goldner-Masson trichrome (GMT) of ctrl and CIA mice; red arrows—areas of infiltration and pannus invasion. **(D)** Flow cytometric analysis of the frequency of granulocytes (Ly6G^+^CD11b^+^) and monocytes (Ly6G^-^CD11b^+^) among CD45^+^ cells released from tarsometatarsal joints of ctrl, CFA, and CIA mice. **(E, F)** Flow cytometric analysis of the frequency of osteoclast progenitor (OCP) populations in periarticular bone marrow (CD45^+^CD3^-^B220^-^DX5^-^Ly6G^-^CD11b^-/lo^CD115^+^, PBM) **(E)** and spleen (CD45^+^CD3^-^B220^-^DX5^-^Ly6G^-^CD11b^+^CD115^+^, SPL) **(F)** in ctrl, CFA, and CIA mice. Representative dot plots are presented. **(G)** Osteoclastogenic potential of PBM and SPL OCPs, stimulated by receptor activator of nuclear factor κB ligand (RANKL) and macrophage colony-stimulating factor (M-CSF), and quantified as the number of tartrate-resistant acid phosphatase (TRAP)^+^ osteoclast-like cells per well (Oc.N.) for ctrl and CIA mice. **(H)** *In vitro* migration assay of sorted PBM OCPs of arthritic mice under the CCR2 small-molecule inhibitor (SMI) treatment, using the Transwell system. Cells migrated to the bottom membrane of the insert were stained with 4’,6-diamidino-2-phenylindole (DAPI), counted and normalized to the average value of non-treated group (NT avg). Cells that migrated to the lower chamber were cultured with RANKL and M-CSF. Representative images of NT, CCL2-, and CCL2/SMI-treated cells (nuclei stained with DAPI) and *in vitro* differentiated TRAP^+^ osteoclast-like cells are presented. The experiments were repeated three times and pooled data are shown (*n* = 6–10 mice per group). Values are presented as medians (middle horizontal lines), boxes represent the interquartile range (IQR), whiskers represent 1.5 times the IQR, and outliers are represented by circles. Statistically significant difference was determined at *p* < 0.05, Mann–Whitney *U*-test **(G)** or Kruskal–Wallis test (*p*-value marked on the graph) with Conover *post-hoc* test for group-to-group comparisons **(B, D, E, F, H)**; correction for multiple comparisons **(D)** was performed using Holm–Bonferroni method; line denotes significant difference between groups.

The final goal of the study was to test if the preventive blockade of CCL2/CCR2 signal could affect the migration of OCPs *in vivo* and mitigate the onset of CIA. However, to translate to the *in vivo* setting, we had to refine the procedure of CCR2 blocking and the mouse model of CIA. We initially planned to apply siRNA as a method of CCR2 knockdown. However, *in vitro* CCR2 siRNA application by electroporation to sorted OCPs was associated with significant cytotoxicity, whereas *in vivo* CCR2 siRNA injections had no effect on CCR2 transcription in OCPs or the course of arthritis (not shown). Therefore, we used CCR2 SMI, shown to be effective in an *in vitro* model (see **Figure 4**), in a dose and schedule published for *in vivo* CCR2 blocking (42, 43). Another modification was the usage of DBA mouse strain for CIA instead of B6 (**Figure 7A**). The rationale behind it was the fact that incidence of CIA in B6 strain is about 50%–60% at best, as opposed to 90%–100% in DBA, consistently. Since we applied the preventive treatment to block the homing of circulatory OCPs and suppress bone resorption (also in accord to 3R principles to reduce number of mice per group), the model with high (nearly 100%) incidence was indispensable.

The pathological findings of bone resorption and inflammatory cell infiltration in the course of CIA developed in the DBA strain were comparable with B6 (**Figures 7B, C**). DBA mice with fully developed CIA had significant loss of talar bone volume and increased proportion of granulocytes and monocytes in the periarticular tibiotalar region (**Figures 7B, D**). The frequency of OCPs was increased in PBM and SPL, with a specifically induced CCR2^hi^ OCP subset compared to control and adjuvant-treated mice (**Figure 7E** for PBM and **Figure 7F** for SPL). Both subsets (CCR2^hi^ and CCR2^lo^) exhibited osteoclastogenic potential similar to the B6 model (**Supplementary Figure 6**), with a significantly larger number of TRAP^+^ osteoclasts generated *in vitro* from PBM and SPL OCPs of arthritic compared to control mice (**Figure 7G**). Finally, using the Transwell culture system, we proved that CCL2-chemotactic gradient enhanced migration of OCPs, and this effect was blocked by CCR2 SMI. Enhanced migration was detected as higher number of cells on the outer side of Transwell membrane and multinucleated osteoclasts in the lower chamber following culture in medium supplemented with RANKL and M-CSF (**Figure 7H**).

**Figure 8:**
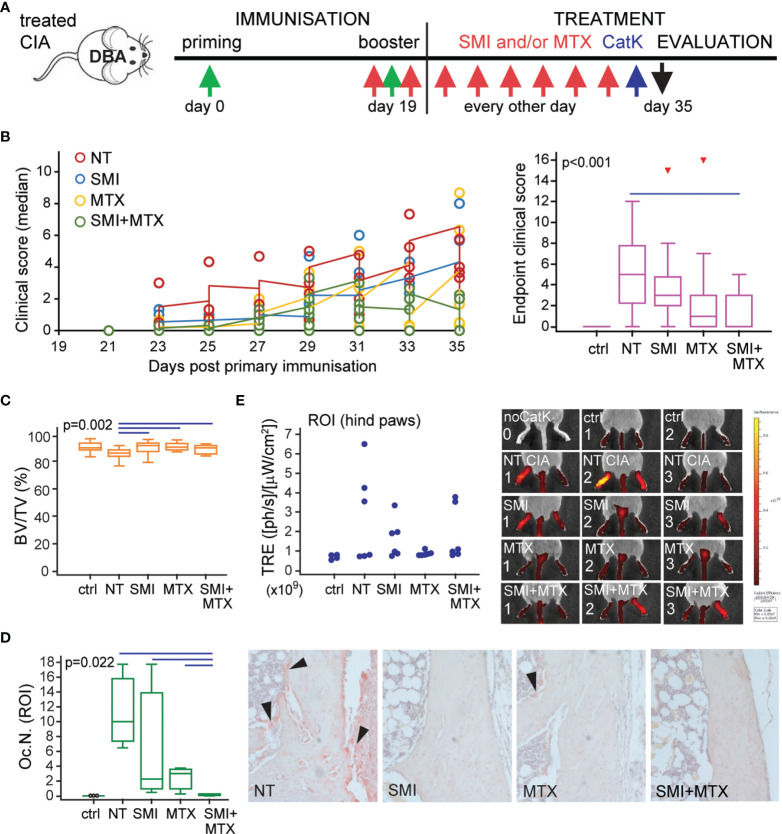
Small-molecule inhibitor of CCR2 (SMI) alone or in combination with methotrexate (MTX) contributes to reduced clinical score and bone resorption in DBA mice with collagen-induced arthritis (CIA). **(A)** Experimental timeline for CIA model and preventive treatment in DBA mice. Starting approximately at day 19 (± 2 days) after the primary immunization, asymptomatic DBA mice were allocated to experimental groups [non-treated (NT) CIA, SMI CIA, MTX CIA, or combined SMI+MTX CIA] and treated every 48 h by intraperitoneal injections. Non-arthritic group was included as control (ctrl). **(B)** Follow-up of the disease clinical scores (left), presented as medians per group for each experiment (total of six experiments) and statistical analysis of clinical score values at experiment endpoint (right) for ctrl, NT, and treated groups. Pooled data from six independent experiments are shown (left, each circle represents median of *n* = 3–4 mice per group per experiment). **(C)** Micro-computed tomography (µCT) analysis of talar bone volume [total bone volume/total tissue volume, BV/TV (%)] for ctrl, NT, and treated groups. **(D)** Number of tartrate-resistant acid phosphatase (TRAP)^+^ osteoclasts (Oc.N.) present in distal tibia endosteal surfaces (region of interest, ROI) for ctrl, NT, and treated groups. Representative images of distal tibia sections histochemically stained for TRAP activity are presented (magnification 100×); black arrows–areas with TRAP^+^ osteoclasts. **(E)** Two-dimensional epifluorescence imaging of CatK 680 FAST probe activation in region of interest (ROI, intact hind paws). Data from one experiment (*n* = 3 mice per group) are shown, and each dot represents the signal intensity measured in a single hind paw. Representative images of IVIS Spectrum epifluorescence imaging of CatK 680 FAST probe signal 24 h after intraperitoneal administration; noCatK—ctrl mouse that did not receive CatK probe. For **(B)** (right), **(C, D)**, values are presented as medians (middle horizontal lines), boxes represent the interquartile range (IQR), whiskers represent 1.5 times the IQR, and outliers are represented by triangles. Statistically significant difference was determined at *p* < 0.05, Kruskal–Wallis test (*p*-value marked on the graph) with Conover *post-hoc* test for group-to-group comparisons; line denotes significant difference between groups. TRE, total radiant efficiency; ph, photon.

Preventive treatment of DBA mice consisted of CCR2 SMI and MTX, applied individually or in combination, and evaluated in comparison to non-treated (NT) mice with CIA and control non-arthritic mice (ctrl). Treatment started immediately prior to secondary immunization to asymptomatic mice and was applied every second day for 2 weeks (**Figure 8A**). A total of six independent experiments (*n* = 3–4 mice per group) were performed for different outcomes to evaluate the severity of arthritis and osteoclast activity. **Figure 8B** includes follow-up of clinical scores (left), presented as medians per group for each experiment (total of six experiments) and statistical analysis of clinical score values at the experiment endpoint (right). Individual treatments delayed the onset of symptoms compared to NT arthritic mice, but only the combined treatment had the protective effect on arthritis development and significantly reduced the clinical score values at the experiment endpoint (**Figure 8B**). Arthritis-induced bone loss was further confirmed by µCT analysis of talar bone volume (**Figure 8C**) and TRAP^+^ osteoclast quantification on histological sections (**Figure 8D**). Both SMI and MTX treatments were able to suppress arthritis-induced bone loss (**Figure 8C**), whereas the combined treatment significantly reduced the number of TRAP^+^ osteoclasts at the tibiotalar joint compared to NT arthritic mice and to both individual treatments (**Figure 8D**).

Osteoclast activity was further evaluated by imaging of activated CatK probe epifluorescence using the IVIS spectrum instrument (44). In the single experiment (*n* = 3 mice per group), mice were injected 24 h prior to sacrifice and imaged at the whole-animal level, followed by imaging of dissected hind paws with the skin removed to eliminate skin-related autofluorescence. Imaging revealed an increased activation of the CatK probe in the affected hind paws, more specifically in the tarsometatarsal region and distal tibia of mice with CIA. Treatments showed a trend of reduced activation of CatK by functional osteoclasts, estimated by scanning of intact mice (**Figure 8E**) or dissected hind limbs (not shown) with similar results. However, due to high individual variability, asymmetric arthritis, low number of included animals, as well as method limitation in precise measurement of fluorescence signal, we presented the obtained results as individual values without further statistical interpretation.

### Preventive *In Vivo* Treatment With CCR2 SMI Suppresses the Accumulation of OCPs in the Periarticular Region of Arthritic Mice

**Figure 9:**
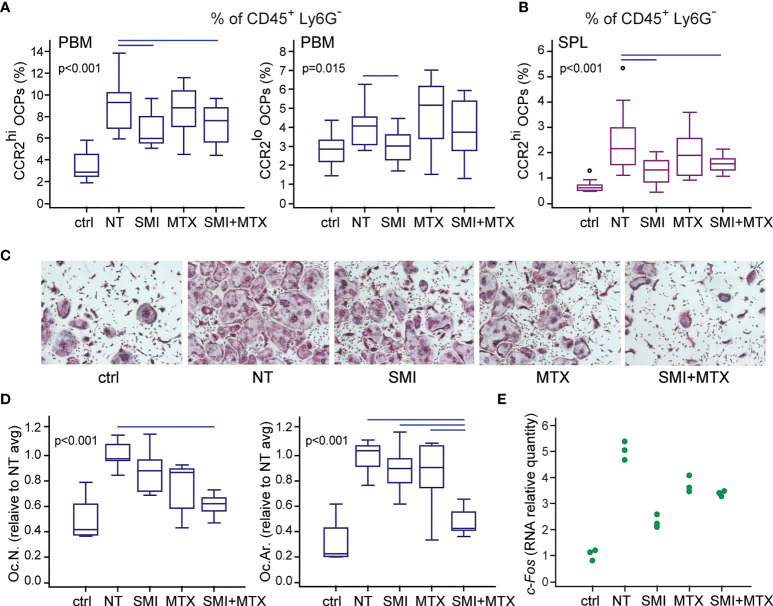
Small-molecule inhibitor of CCR2 (SMI) alone or in combination with methotrexate (MTX) contributes to reduced osteoclast progenitor (OCP) frequency and their lower osteoclastogenic potential in DBA mice with collagen-induced arthritis (CIA). Starting approximately at day 19 (± 2 days) after the primary immunization, asymptomatic DBA mice were allocated to experimental groups [non-treated (NT) CIA, SMI CIA, MTX CIA, or combined SMI+MTX CIA] and treated every 48 h by intraperitoneal injections. Non-arthritic group was included as control (ctrl). **(A)** Flow cytometric analysis of the frequency of OCPs in periarticular bone marrow (PBM) bearing the phenotype CD45^+^CD3^-^B220^-^DX5^-^Ly6G^-^CD11b^-/lo^CD115^+^CCR2^hi^ and CD45^+^CD3^-^B220^-^DX5^-^Ly6G^-^CD11b^-/lo^CD115^+^CCR2^lo^. **(B)** Flow cytometric analysis of the frequency of OCPs in spleen (SPL), bearing the phenotype CD45^+^CD3^-^B220^-^DX5^-^Ly6G^-^CD11b^+^CD115^+^CCR2^hi^. **(C)** Sorted PBM OCPs (CD45^+^CD3^-^B220^-^DX5^-^Ly6G^-^CD11b^-/lo^CD115^+^) from *in vivo* treated mice were seeded to plates and stimulated by receptor activator of nuclear factor κB ligand (RANKL) and macrophage colony-stimulating factor (M-CSF). Representative microphotographs of *in vitro* differentiated tartrate-resistant acid phosphatase (TRAP)^+^ osteoclast-like cells are presented (magnification 100×). **(D)** Quantification of the osteoclastogenic potential of PBM OCPs from treated mice. Culture-differentiated multinuclear TRAP^+^ osteoclast-like cells per well (Oc.N.) and percentage of TRAP^+^ area per well (Oc.Ar.) were calculated and normalized to the average value of NT group (NT avg). **(E)** qPCR analysis of *c-Fos* gene expression in cultures of OCPs from treated mice, stimulated by RANKL and M-CSF for 2 days, presented as relative quantity of mRNA. Dots represent technical replicates from single experiment. For **(A, B, D)** experiments were repeated three times and pooled data are shown (*n* = 10–12 mice per group). Values are presented as medians (middle horizontal lines), boxes represent the interquartile range (IQR), whiskers represent 1.5 times the IQR, and outliers are represented by circles. Statistically significant difference was determined at *p* < 0.05, Kruskal–Wallis test (*p*-value marked on the graph) with Conover *post-hoc* test for group-to-group comparisons; line denotes significant difference between groups.

Since the preventive treatment with CCR2 SMI alone or in combination with MTX showed preferable effects on the reduction of bone resorption in arthritis, we next examined its effect on myeloid cell accumulation in the periarticular region of arthritic mice after *in vivo* treatment by the same scheme as described in **Figure 8**. Individual and combined treatment by CCR2 SMI significantly reduced accumulation of CCR2^hi^ OCPs in PBM compared to NT arthritic mice (**Figure 9A**). The same effect of CCR2 SMI (applied alone or in combination) on the reduction of CCR2^hi^ OCP frequency was observed in SPL (**Figure 9B**), possibly indicating that the CCL2/CCR2 axis is not only important for OCP migration, but also for the commitment of extramedullary reservoir of myeloid progenitors toward osteoclast lineage. In addition, overall frequencies of total granulocyte (CD45^+^Ly6G^+^), monocyte (CD45^+^Ly6C^+^), and macrophage (CD45^+^F4/80^+^) populations were reduced in periarticular regions (PBM and TMT) after combined CCR2 SMI and MTX treatment compared to NT arthritic mice. The effect was even more pronounced in SPL, where all treated groups were significantly different from NT arthritic mice for granulocyte and monocyte populations (**Supplementary Figure 7**).

Finally, FACS-sorted and cultured periarticular OCPs from mice treated by the combination of CCR2 SMI and MTX exhibited significantly suppressed osteoclastogenic potential in cultures stimulated by RANKL and M-CSF, detected as a reduced number of multinucleated TRAP^+^ osteoclasts (**Figures 9C, D**). Importantly, the area covered by differentiated osteoclasts was significantly lower (implying smaller osteoclasts) in combined compared to both individual treatments, indicating the preferable anti-osteoclastogenic effect of drug combination (**Figure 9D**). At the level of gene expression, OCPs from mice treated *in vivo*, and stimulated by RANKL and M-CSF *in vitro*, exhibited trend of *c-Fos* downregulation, required for osteoclast differentiation (38). Since *c-Fos* was evaluated in a single experiment (pooled RNA per group), data are presented as individual values of technical replicates without further statistical interpretation (**Figure 9E**).

Collectively, data obtained by *in vivo* treatment provide the functional confirmation of the ability of complementary treatment by CCL2/CCR2 axis blocking, in addition to MTX as a commonly recommended DMARD for RA treatment, to reduce osteoclast number and activity and therefore protect mice from arthritis-induced bone loss.

## Discussion

Osteoclasts are cells of hematopoietic origin with the unique ability to affect skeletal metabolism through bone resorption (45). Due to their myeloid origin, they are induced by the autoinflammatory/autoimmune response, contributing to the bone pathology of immune-mediated diseases (2, 38, 46). In this study, we identified the OCP subset with a high level of CCR2 expression that is specifically induced by the mouse model of RA, being present in SPL (CD45^+^Ly6G^-^CD3^-^B220^-^NK1.1^-^CD11b^+^CD115^+^CCR2^hi^) and PBM (CD45^+^Ly6G^-^CD3^-^B220^-^NK1.1^-^CD11b^-/lo^CD115^+^CCR2^hi^). This subpopulation is highly migratory toward the chemokine gradient, particularly CCL2 signal, and is able to home to the inflamed joints. We also identified a similar CCR2^hi^ subset of attractable circulatory OCPs existing in human PBL of patients with RA. Approved treatments for RA mostly include standard or biological drugs that affect the inflammatory arm, without precise characterization of their action on osteoclasts, as crucial effector cells causing bone destruction and irreversible joint damage. We propose that addition of CCR2 blocking to standard treatment, i.e., DMARDs, early in the course of arthritis may significantly improve disease outcome by suppressing infiltration of circulatory OCPs and, thus, reduce the number of active bone-resorbing osteoclasts.

Inflammatory cell infiltrate replacing steady-state bone marrow composition has long been observed in mouse and human arthritis (47, 48). Among infiltrating cells in periarticular compartments (distal tibia PBM and hind paw TMT small joints), we observed an increased frequency of highly osteoclastogenic OCPs, but, in addition, the arthritis-associated subset of OCPs was enlarged in SPL and PBL. Detailed characterization of medullary and extramedullary reservoirs of OCPs is required to understand the pathophysiology of increased osteoclast activity causing periarticular and systemic bone resorption in arthritis (38, 46). The question whether infiltrating OCPs are committed progenitors of bone marrow origin or whether they are the progeny of cells released from extramedullary reservoirs is still a matter of debate. Jacome-Galarza et al. precisely described distinct phenotype of bone marrow versus spleen OCPs, indicating that the transition from bone marrow to peripheral progenitors is accompanied by phenotypic changes (acquisition of CD11b) (6). However, they proposed that circulating OCPs are derived from common bone marrow osteoclast/macrophage/dendritic cell progenitor. In contrast, Yahara et al. suggested that spleen erythromyeloid progenitor-derived yolk sac OCPs arise independently from hematopoietic-stem cell lineage and provide osteoclasts that contribute to physiological bone remodeling as well as injury-induced remodeling (8). In the CIA model, we identified significantly expanded population of CCR2^hi^ OCPs, of both bone marrow- and circulatory-like phenotype (CD11b^-/lo^ and CD11b^+^, respectively). In control mice, the entire peripheral OCP population (in SPL and PBL) coexpresses CCR2 and CX3CR1, whereas in arthritic mice, a small population of CCR2^+^CX3CR1^-^ cells is detectable, besides the larger CCR2^+^CX3CR1^+^ subset. This is in line with the studies assigning CX3CR1^lo^CCR2^+^ cells to inflammatory monocytes, susceptible to prompt mobilization to the site of inflammation (49, 50). Distal tibia PBM of arthritic mice also contains a CCR2^hi^ subset of OCPs (corresponding to 2%–3% of total CD45^+^ cells), but, unlike SPL, includes a smaller population of CCR2^lo^ cells (corresponding to less than 1% of total CD45^+^ cells). Both of these subsets possess high osteoclastogenic potential and bone-resorbing activity, but differ in morphology and phenotype properties. CCR2^lo^ cells are higher on FSC/SSC parameters, and mostly CD11b^-^, with potent proliferative capacity, whereas CCR2^hi^ cells are smaller, with a low level of CD11b expression and slow cell-cycling behavior. Comparative osteoclastogenic cultures of CCR2^+^CX3CR1^+^ and CCR2^+^CX3CR1^-^ subsets proved that CX3CR1 expression is not essential for the osteoclastogenic potential, since CCR2^+^CX3CR1^-^ cells produced an even higher number of TRAP^+^ osteoclasts compared with the same-density cultures of the CCR2^+^CX3CR1^+^ subset. Similar to spleen OCPs, the periarticular CCR2^hi^ subset is strongly induced by arthritis, which prompts us to hypothesize that these OCPs may be the infiltrating population attracted through the CCL2 signal from the circulatory pool released by spleen. This notion is supported by the finding of strong CCL2 protein expression in multiple areas of inflamed joints, including bone marrow adjacent to active osteoclasts attached to bone surface. This population fits the described properties of peripheral monocytes, as cells with slow proliferative capacity and more mature phenotype (6, 51). On the other side, expansion of CCR2^lo^ subset is less prominent and may be ascribed to inflammation, observed in adjuvant-treated and arthritic groups. Moreover, gene expression analysis of cultured OCPs showed that osteoclast-differentiation genes (*c-Fos, CD115/c-Fms*, and *Rank*) and chemokine CCL2 are induced in the CCR2^lo^ subset of both adjuvant-treated and arthritic groups, whereas arthritis-specific enhancement was observed in CCR2^hi^ OCPs. Previous mouse and human studies indicated that CCL2 plays a role in immune-cell infiltration of tissues during immune-mediated inflammatory diseases (52, 53) and high stimulation of CCL2 expression in CCR2^hi^ OCPs by arthritis suggests that it can act in an autocrine manner on osteoclast lineage cells (52, 54). We may speculate that inflammation alone is sufficient to expand immature myeloid cells, but autoimmune response is required to mobilize spleen reservoir and attract circulatory OCPs to the inflamed joints.

Several important studies emerged recently to address the relative contribution of circulatory versus bone marrow OCP pool to bone remodeling in basal and induced conditions. Our study supports the hypothesis that infiltrating OCPs may be of the spleen origin. We showed expansion of total population of CD11b^+^ spleen cells as well as the frequency of CD45^+^CD3^-^B220^-^NK1.1^-^CD11b^+^CD115^+^ cells, indicating significantly enlarged pool of peripheral OCPs. Joint inflammation is accompanied by high local production of cytokines and chemokines, profuse vascularization, and tissue swelling (1). *In vivo* intravascular staining showed a higher frequency of OCPs in the periarticular area of arthritic mice compared to controls. In addition, adoptively transferred tdTomato-positive spleen OCPs were able to home to periarticular area of affected joints. Still, there is a puzzle of phenotypic difference between circulatory and medullary OCPs and functional relation between these subsets. Integrin CD11b is highly expressed by circulatory CCR2^hi^ OCPs, whereas the corresponding CCR2^hi^ bone marrow OCPs only weakly express this integrin. We observed that adoptively transferred CD11b^+^tdTomato^+^ OCPs reduce the level of CD11b expression upon homing to the periarticular bone marrow (not shown). Also, peripheral CD11b^+^ OCPs downregulated CD11b upon short-term osteoclastogenic culture (not shown). Therefore, we believe that population of circulatory OCPs, originating from spleen reservoir, downregulates CD11b upon homing to the bone surface, as known for integrin dynamic expression in inflammatory conditions (6, 55, 56). In particular, inflammatory environment in arthritis is increasingly supportive for peripheral myeloid cell attraction, including OCPs. Involvement of peripheral OCPs in other pathologic conditions, such as bone fracture, was recently shown by Novak et al. (12), contrasting findings by Jacome-Galarza et al., who implicated the predominance of bone-marrow-derived progenitor differentiation (51).

Present therapeutic approaches to RA include early and continuous medication to achieve remission using DMARDs such as MTX and biologic anticytokine therapies (57). Anti-inflammatory therapy indirectly decreases osteoclast activation, rather than directly interfering with their action. The precise mechanism of MTX action in RA is not known (58), but demonstrated clinical efficacy makes MTX the number one choice of therapy in good responders. Its effect on bone homeostasis at low-dose treatment as in RA is also unclear, while, with dose increment, there is a risk of MTX osteopathy (59). Despite intervention, some patients fail to respond to medication, and even minimally active disease promotes further local and systemic bone loss (60, 61). So far, conducted clinical trials failed to demonstrate an effect of CCR2/CCL2 blocking antibodies in RA (23, 24), which may be due to numerous reasons (62); however, the research duration was short and focused on inflammation dampening outcomes, and the potential beneficial effects on bone were not evaluated. In our study, after demonstrating that mouse and human OCPs can respond to CCL2 chemokine gradient, we assessed the impact of *in vivo* CCR2 blockade by SMI on bone resorption. Early time point (as the preventive treatment) was selected to forestall inflammatory infiltrate formation in the subchondral and periarticular bone. Alongside reducing clinical symptoms of arthritis and synovitis, as already described for CCR2/CCL2 blockade (20), SMI alone or in combination with MTX showed a decrease in bone loss, and the reduction of myeloid lineage cell accumulation locally and in spleen. Moreover, only the combined treatment decreased OCP recruitment to inflamed joints, simultaneously also diminishing their osteoclastogenic potential, compared to individual drugs or non-treated arthritis. Therefore, we suggest revising the CCR2 targeting by SMI in RA treatment (63). Furthermore, using a SMI over neutralizing antibodies includes the possibility of oral administration, which, in regard to patient compliance, should not be neglected (64).

## Conclusions

Despite the undeniable fact that peripheral OCPs exist in physiological and pathological conditions, they could not differentiate into mature osteoclasts unless attracted to the bone surface. Their plasticity and migratory ability make them appropriate for prompt mobilization upon stimulation, including inflammation. However, data on their functional capability and signals for their attraction in different pathological conditions are still limited. We identified the CCR2^hi^ OCP subset, specifically induced in arthritis, with high osteoclastogenic potential. Moreover, *in vivo* CCR2 SMI blockade in combination with MTX was able to decrease disease clinical score and osteoclast resorptive activity as well as OCP frequency and differentiation potential. Novel therapies, such as biologicals and JAK inhibitors, have halted radiographic progression in most, but not all, of the patients (65), so there is still a need for investigation of alternative treatment modes. Based on the proven importance of the CCL2/CCR2 axis in mouse and human OCP migration, we propose the addition of CCL2/CCR2 blockade early at the course of arthritis as a promising approach to reduce osteoresorptive damage.

## Data Availability Statement

The original contributions presented in the study are included in the article/**Supplementary Material**. Further inquiries can be directed to the corresponding author.

## Ethics Statement

The studies involving human participants were reviewed and approved by the Ethics Committee of the University of Zagreb, School of Medicine, Zagreb, Croatia. The patients/participants provided their written informed consent to participate in this study. The animal study was reviewed and approved by Ministarstvo poljoprivrede (Ministry of Agriculture), Uprava za veterinarstvo i sigurnost hrane, Zagreb, Croatia, and the Ethics Committee of the University of Zagreb, School of Medicine, Zagreb, Croatia.

## Author Contributions

DF, DG, MF, and AŠ designed the study. DF, DG, MF, AŠ, AM, NL, DŠ, MIM, NK, ZJ, VK, and TK performed the experiments. DF, DG, MF, AŠ, AM, NL, DŠ, MIM, NK, ZJ, VK, and TK acquired and analyzed data. DF, DG, MF, and AŠ interpreted the results. DF, DG, MF, AŠ, and NK prepared the manuscript. All authors critically revised the manuscript and approved the final version.

## Funding

This work was supported by grants from the Croatian Science Foundation (IP-2018-01-2414, DOK-2018-09-4276 and UIP-2017-05-1965), by the Scientific Center of Excellence for Reproductive and Regenerative Medicine, Republic of Croatia, and by the European Union through the European Regional Development Fund, under grant agreement No. KK.01.1.1.01.0008, project “Reproductive and Regenerative Medicine - Exploring New Platforms and Potentials”.

## Conflict of Interest

The authors declare that the research was conducted in the absence of any commercial or financial relationships that could be construed as a potential conflict of interest.

## Publisher’s Note

All claims expressed in this article are solely those of the authors and do not necessarily represent those of their affiliated organizations, or those of the publisher, the editors and the reviewers. Any product that may be evaluated in this article, or claim that may be made by its manufacturer, is not guaranteed or endorsed by the publisher.

## Abbreviations

CIA, Collagen-induced arthritis; M-CSF, Macrophage Colony-stimulating Factor; MTX, methotrexate; μCT, micro-computerized tomography; NT, non-treated; OCP, osteoclast progenitor; PBM, periarticular bone marrow; RA, Rheumatoid arthritis; RANKL, Receptor activator of NF-κB ligand; SMI, small-molecule inhibitor; SPL, spleen; TMT, tarsometatarsal; TRAP, tartrate-resistant acid phosphatase.
