# Supplementary material for: Preventive CCL2/CCR2 Axis Blockade Suppresses Osteoclast Activity in a Mouse Model of Rheumatoid Arthritis by Reducing Homing of CCR2hi Osteoclast Progenitors to the Affected Bone
Source: Front Immunol. 2021 Dec 3;12:767231. doi: 10.3389/fimmu.2021.767231 (PMC8677701; doi:10.3389/fimmu.2021.767231)
Supplement: Supplementary file 1 [file Presentation_1.pptx]

## Slide 1
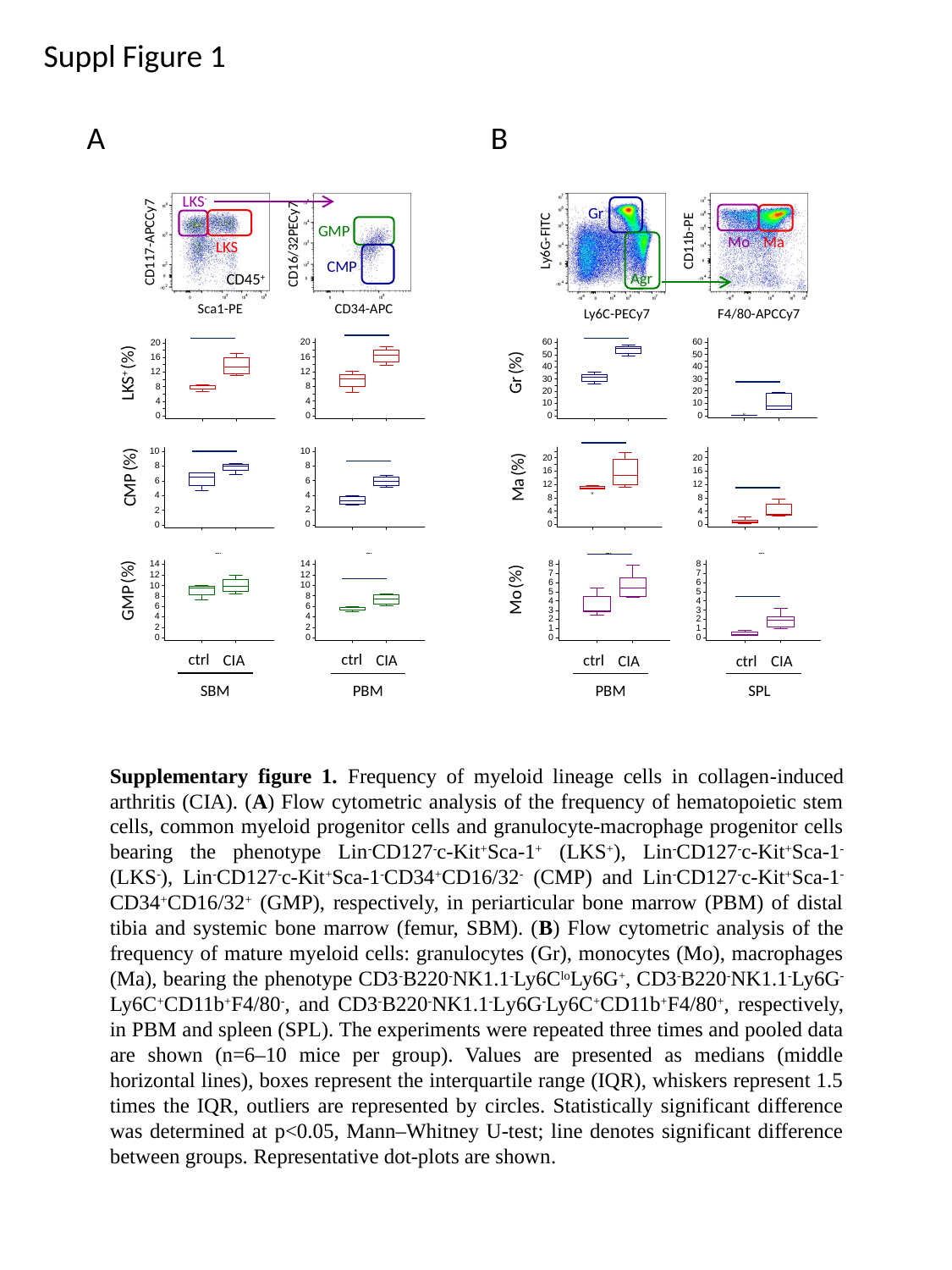

Suppl Figure 1
A
B
LKS-
Gr
GMP
Mo
Ma
Ly6G-FITC
CD11b-PE
CD117-APCCy7
CD16/32PECy7
LKS
CMP
Agr
CD45+
Sca1-PE
CD34-APC
Ly6C-PECy7
F4/80-APCCy7
Gr (%)
LKS+ (%)
Ma (%)
CMP (%)
Mo (%)
GMP (%)
ctrl
ctrl
CIA
ctrl
CIA
ctrl
CIA
CIA
SBM
PBM
PBM
SPL
Supplementary figure 1. Frequency of myeloid lineage cells in collagen-induced arthritis (CIA). (A) Flow cytometric analysis of the frequency of hematopoietic stem cells, common myeloid progenitor cells and granulocyte-macrophage progenitor cells bearing the phenotype Lin-CD127-c-Kit+Sca-1+ (LKS+), Lin-CD127-c-Kit+Sca-1- (LKS-), Lin-CD127-c-Kit+Sca-1-CD34+CD16/32- (CMP) and Lin-CD127-c-Kit+Sca-1-CD34+CD16/32+ (GMP), respectively, in periarticular bone marrow (PBM) of distal tibia and systemic bone marrow (femur, SBM). (B) Flow cytometric analysis of the frequency of mature myeloid cells: granulocytes (Gr), monocytes (Mo), macrophages (Ma), bearing the phenotype CD3-B220-NK1.1-Ly6CloLy6G+, CD3-B220-NK1.1-Ly6G-Ly6C+CD11b+F4/80-, and CD3-B220-NK1.1-Ly6G-Ly6C+CD11b+F4/80+, respectively, in PBM and spleen (SPL). The experiments were repeated three times and pooled data are shown (n=6–10 mice per group). Values are presented as medians (middle horizontal lines), boxes represent the interquartile range (IQR), whiskers represent 1.5 times the IQR, outliers are represented by circles. Statistically significant difference was determined at p<0.05, Mann–Whitney U-test; line denotes significant difference between groups. Representative dot-plots are shown.

## Slide 2
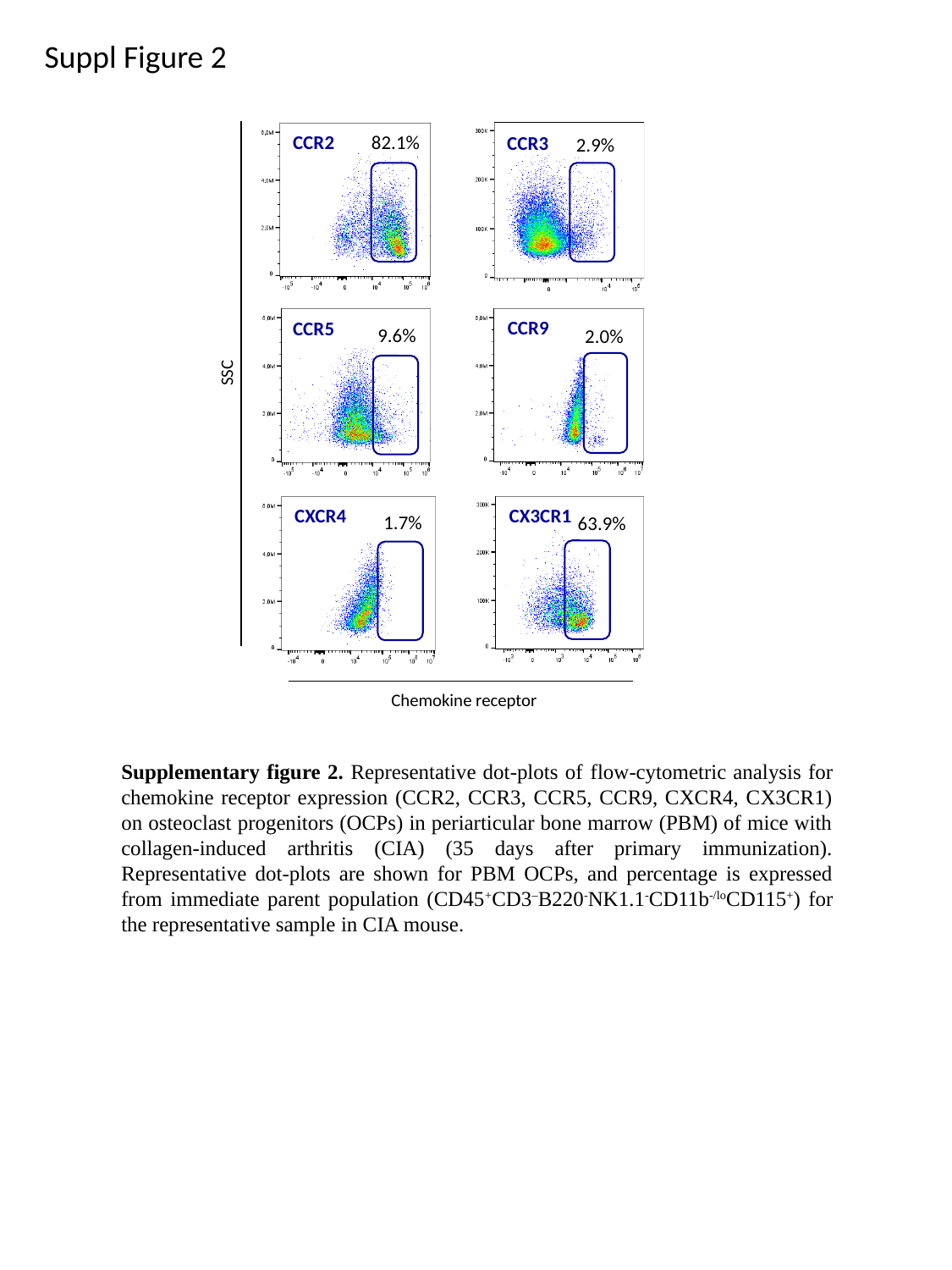

Suppl Figure 2
82.1%
CCR2
CCR3
2.9%
CCR9
CCR5
9.6%
2.0%
SSC
CXCR4
CX3CR1
1.7%
63.9%
Chemokine receptor
Supplementary figure 2. Representative dot-plots of flow-cytometric analysis for chemokine receptor expression (CCR2, CCR3, CCR5, CCR9, CXCR4, CX3CR1) on osteoclast progenitors (OCPs) in periarticular bone marrow (PBM) of mice with collagen-induced arthritis (CIA) (35 days after primary immunization). Representative dot-plots are shown for PBM OCPs, and percentage is expressed from immediate parent population (CD45+CD3–B220-NK1.1-CD11b-/loCD115+) for the representative sample in CIA mouse.

## Slide 3
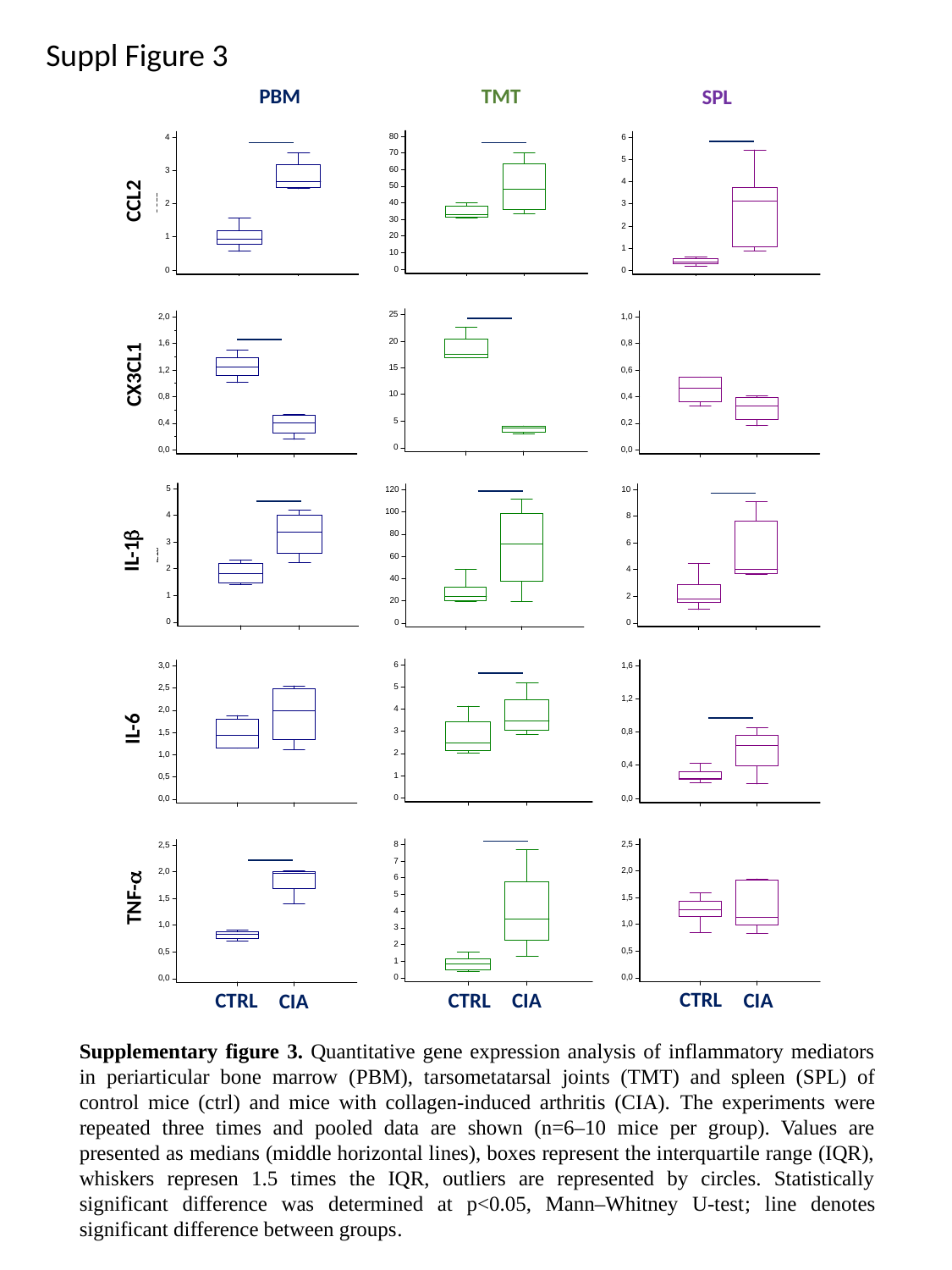

Suppl Figure 3
TMT
PBM
SPL
CCL2
CX3CL1
IL-1b
IL-6
TNF-a
CTRL
CTRL
CIA
CTRL
CIA
CIA
Supplementary figure 3. Quantitative gene expression analysis of inflammatory mediators in periarticular bone marrow (PBM), tarsometatarsal joints (TMT) and spleen (SPL) of control mice (ctrl) and mice with collagen-induced arthritis (CIA). The experiments were repeated three times and pooled data are shown (n=6–10 mice per group). Values are presented as medians (middle horizontal lines), boxes represent the interquartile range (IQR), whiskers represen 1.5 times the IQR, outliers are represented by circles. Statistically significant difference was determined at p<0.05, Mann–Whitney U-test; line denotes significant difference between groups.

## Slide 4
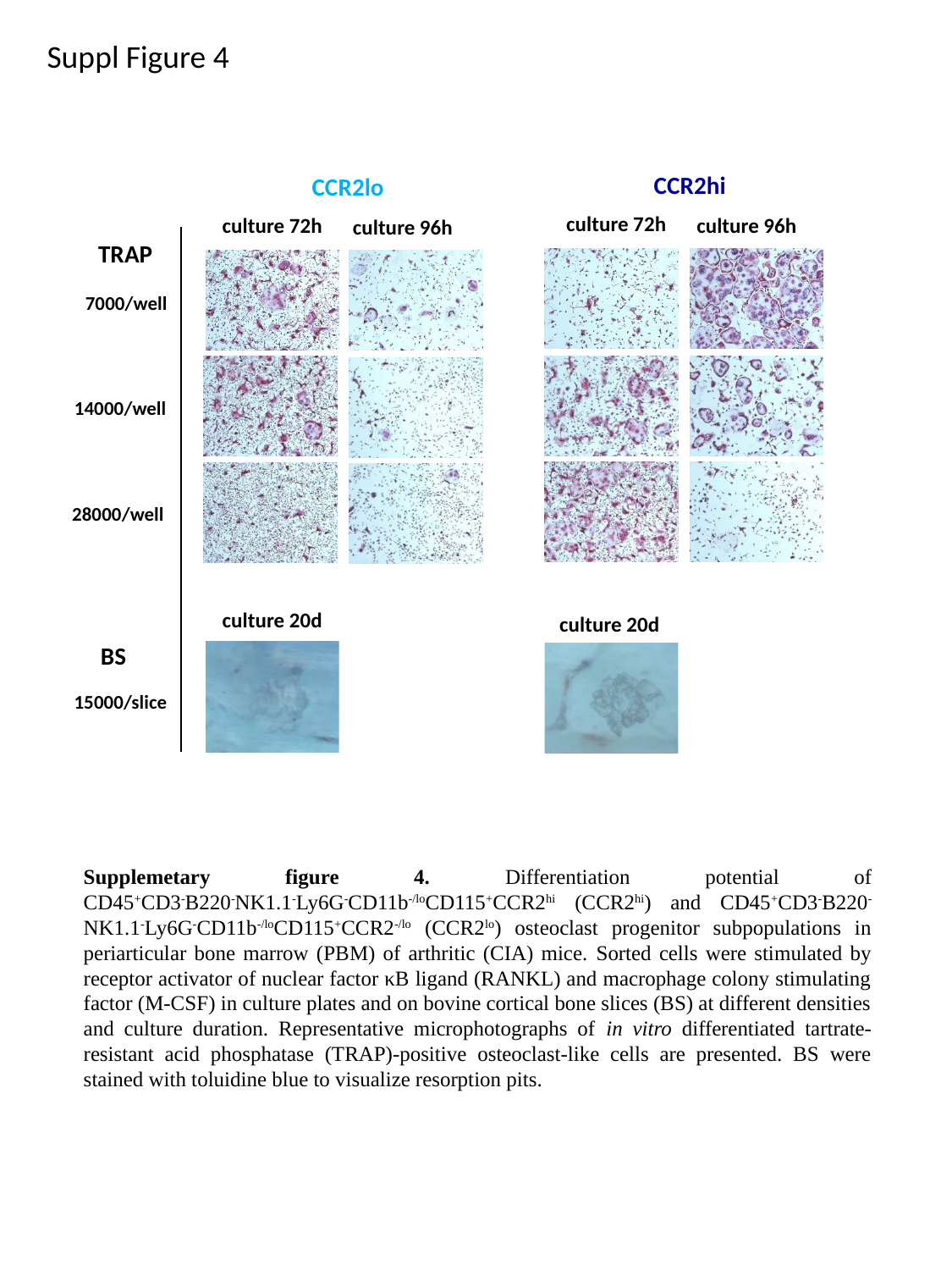

Suppl Figure 4
CCR2hi
CCR2lo
culture 72h
culture 72h
culture 96h
culture 96h
TRAP
7000/well
14000/well
28000/well
culture 20d
culture 20d
BS
15000/slice
Supplemetary figure 4. Differentiation potential of CD45+CD3-B220-NK1.1-Ly6G-CD11b-/loCD115+CCR2hi (CCR2hi) and CD45+CD3-B220-NK1.1-Ly6G-CD11b-/loCD115+CCR2-/lo (CCR2lo) osteoclast progenitor subpopulations in periarticular bone marrow (PBM) of arthritic (CIA) mice. Sorted cells were stimulated by receptor activator of nuclear factor κB ligand (RANKL) and macrophage colony stimulating factor (M-CSF) in culture plates and on bovine cortical bone slices (BS) at different densities and culture duration. Representative microphotographs of in vitro differentiated tartrate-resistant acid phosphatase (TRAP)-positive osteoclast-like cells are presented. BS were stained with toluidine blue to visualize resorption pits.

## Slide 5
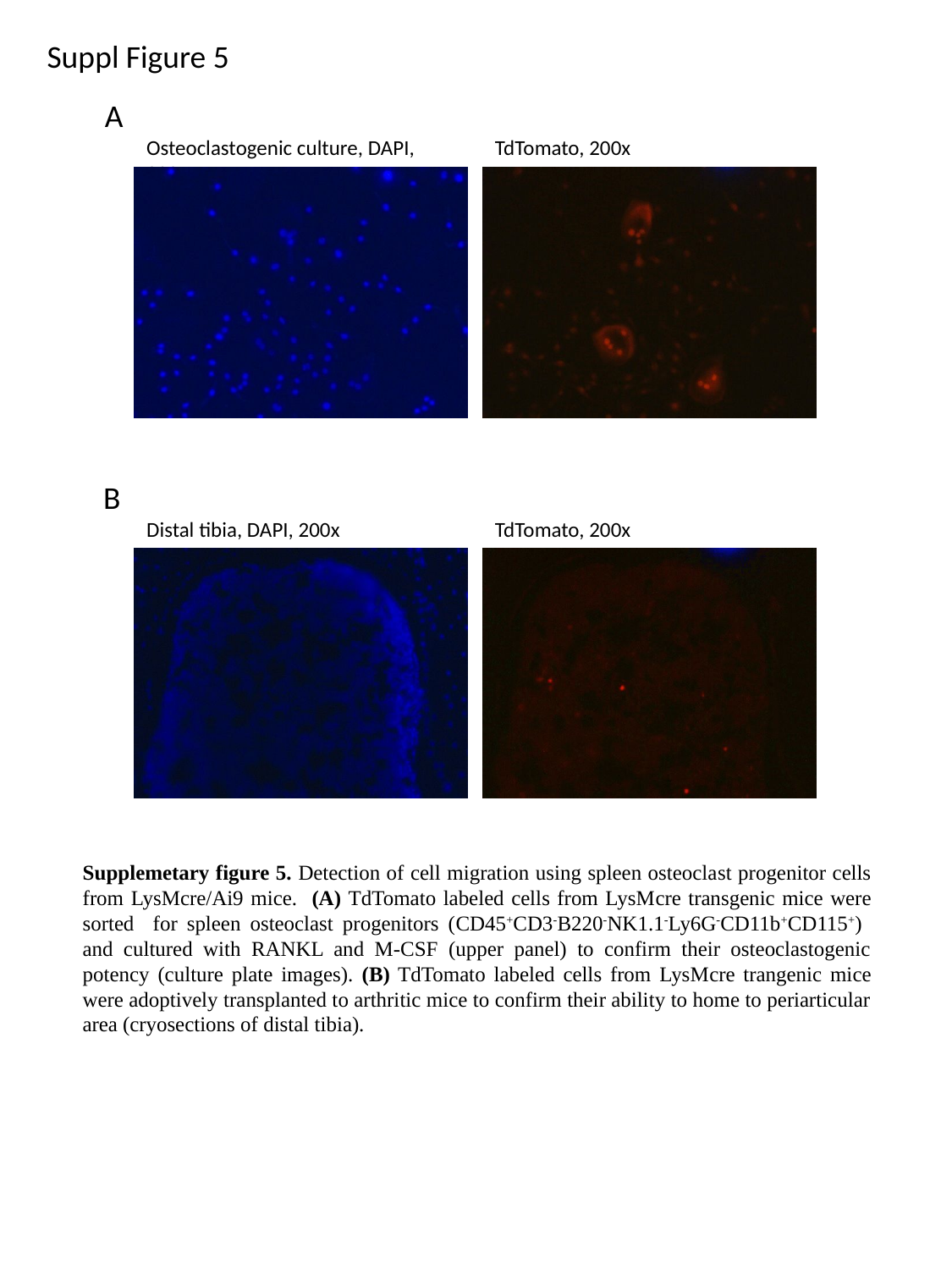

Suppl Figure 5
A
Osteoclastogenic culture, DAPI, 200x
TdTomato, 200x
Distal tibia, DAPI, 200x
TdTomato, 200x
B
Supplemetary figure 5. Detection of cell migration using spleen osteoclast progenitor cells from LysMcre/Ai9 mice. (A) TdTomato labeled cells from LysMcre transgenic mice were sorted for spleen osteoclast progenitors (CD45+CD3-B220-NK1.1-Ly6G-CD11b+CD115+) and cultured with RANKL and M-CSF (upper panel) to confirm their osteoclastogenic potency (culture plate images). (B) TdTomato labeled cells from LysMcre trangenic mice were adoptively transplanted to arthritic mice to confirm their ability to home to periarticular area (cryosections of distal tibia).

## Slide 6
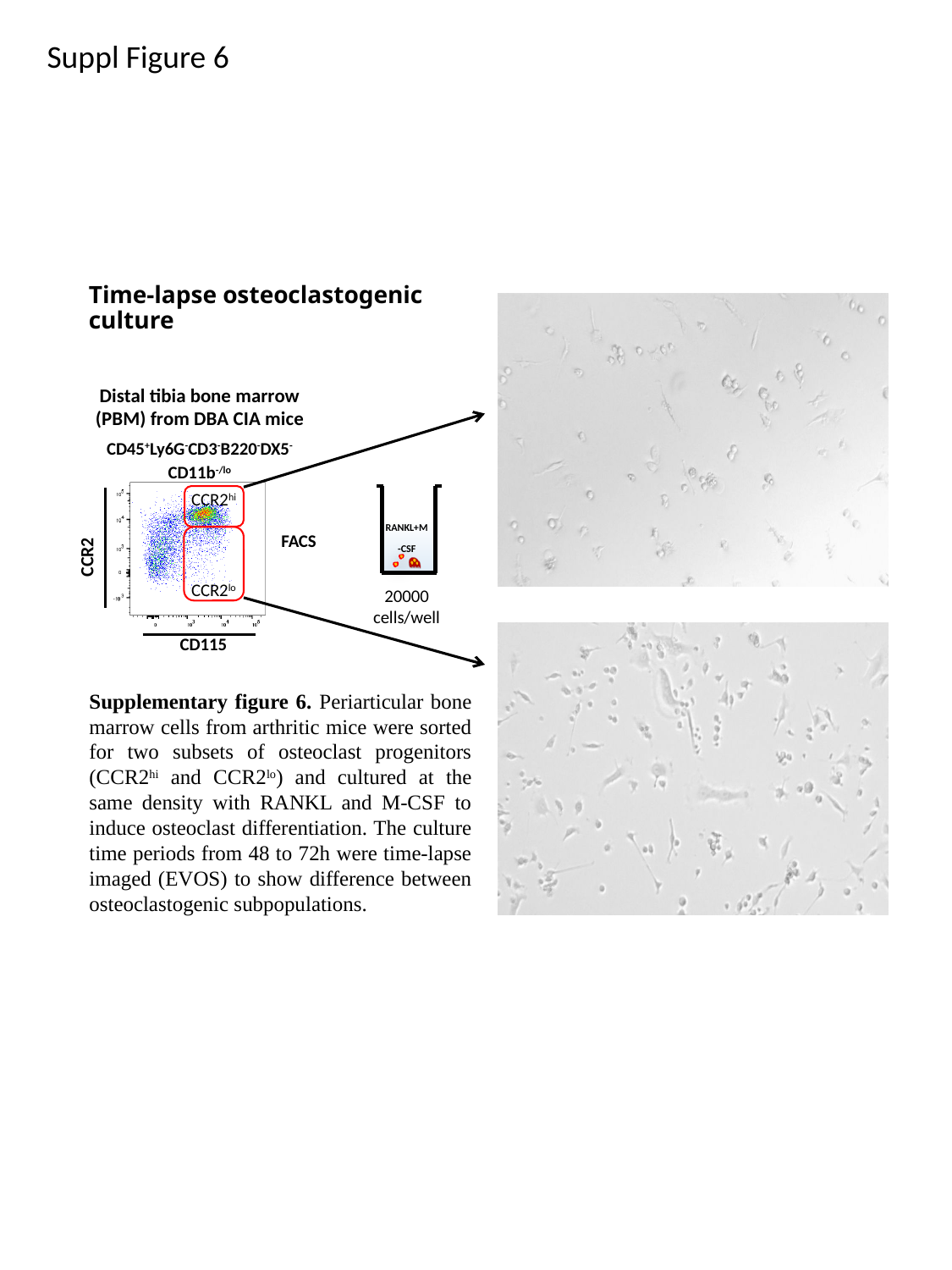

Suppl Figure 6
# Time-lapse osteoclastogenic culture
Distal tibia bone marrow (PBM) from DBA CIA mice
CD45+Ly6G-CD3-B220-DX5- CD11b-/lo
CCR2hi
CCR2
FACS
CCR2lo
RANKL+M-CSF
20000 cells/well
CD115
Supplementary figure 6. Periarticular bone marrow cells from arthritic mice were sorted for two subsets of osteoclast progenitors (CCR2hi and CCR2lo) and cultured at the same density with RANKL and M-CSF to induce osteoclast differentiation. The culture time periods from 48 to 72h were time-lapse imaged (EVOS) to show difference between osteoclastogenic subpopulations.

## Slide 7
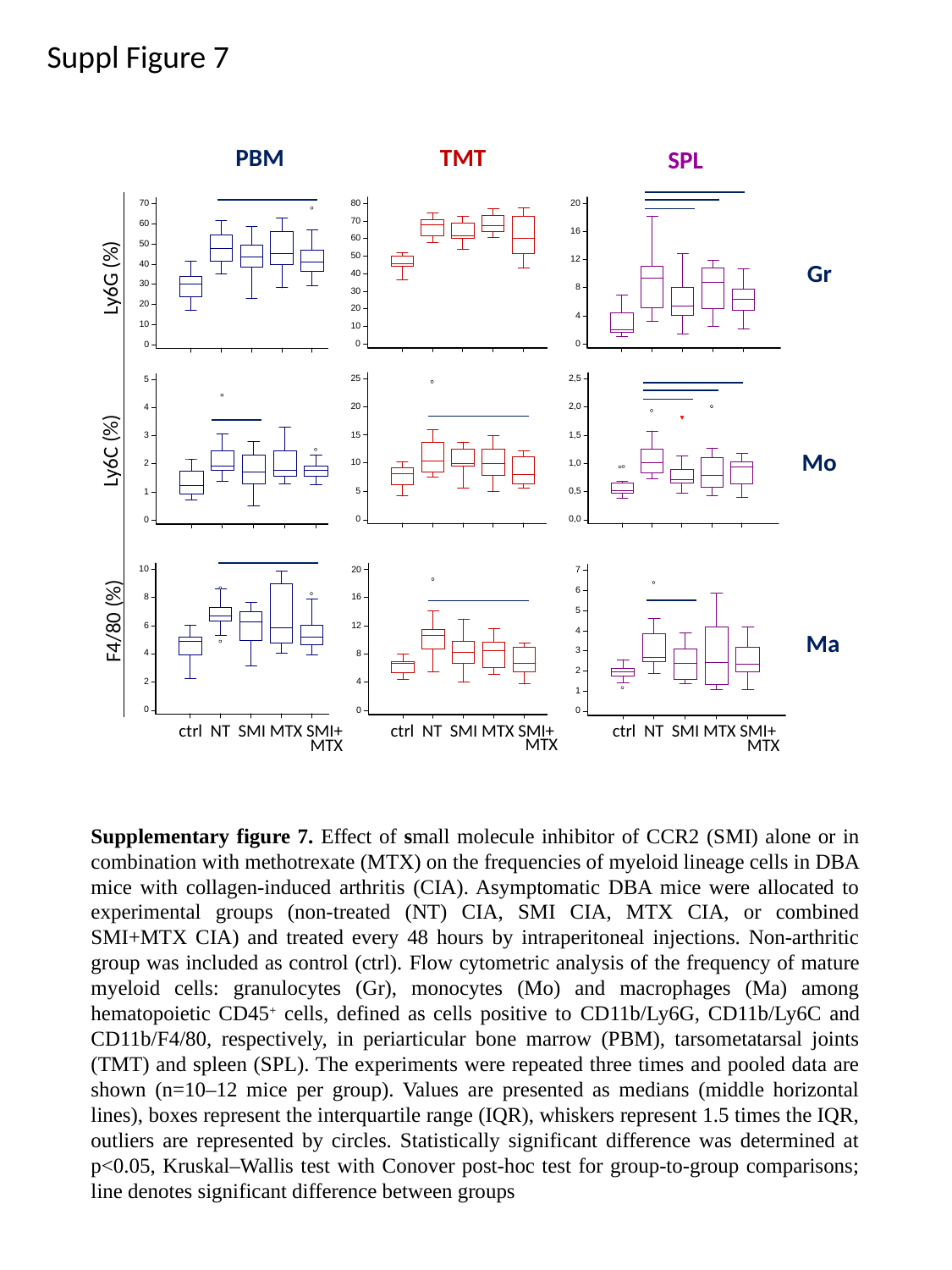

Suppl Figure 7
TMT
PBM
SPL
Gr
Mo
Ma
Ly6G (%)
Ly6C (%)
F4/80 (%)
ctrl NT SMI MTX SMI+
ctrl NT SMI MTX SMI+
ctrl NT SMI MTX SMI+
MTX
MTX
MTX
Supplementary figure 7. Effect of small molecule inhibitor of CCR2 (SMI) alone or in combination with methotrexate (MTX) on the frequencies of myeloid lineage cells in DBA mice with collagen-induced arthritis (CIA). Asymptomatic DBA mice were allocated to experimental groups (non-treated (NT) CIA, SMI CIA, MTX CIA, or combined SMI+MTX CIA) and treated every 48 hours by intraperitoneal injections. Non-arthritic group was included as control (ctrl). Flow cytometric analysis of the frequency of mature myeloid cells: granulocytes (Gr), monocytes (Mo) and macrophages (Ma) among hematopoietic CD45+ cells, defined as cells positive to CD11b/Ly6G, CD11b/Ly6C and CD11b/F4/80, respectively, in periarticular bone marrow (PBM), tarsometatarsal joints (TMT) and spleen (SPL). The experiments were repeated three times and pooled data are shown (n=10–12 mice per group). Values are presented as medians (middle horizontal lines), boxes represent the interquartile range (IQR), whiskers represent 1.5 times the IQR, outliers are represented by circles. Statistically significant difference was determined at p<0.05, Kruskal–Wallis test with Conover post-hoc test for group-to-group comparisons; line denotes significant difference between groups
